# Immune Infiltration‐Related Genes as Potential Biomarkers and Predicted Targets for Renal Allograft Delayed Graft Function and Survival Outcome: An Integrated Machine Learning Approach and Drugs Analysis

**DOI:** 10.1155/mi/1451740

**Published:** 2026-06-12

**Authors:** Yifei Zhang, Yuqing Li, Xuemeng Qiu, Jiyue Wu, Qing Bi, Peng Cao, Jiandong Zhang, Wei Wang

**Affiliations:** ^1^ Department of Urology, Beijing Chao-Yang Hospital, Capital Medical University, Chaoyang District, Beijing, 100020, China, ccmu.edu.cn; ^2^ Institute of Urology, Capital Medical University, Fengtai District, Beijing, 100071, China, ccmu.edu.cn

**Keywords:** biomarkers, delayed graft function, drug prediction, immune cell infiltration, ischemia-reperfusion injury, kidney transplant, machine learning

## Abstract

**Background:**

Ischemia‐reperfusion injury (IRI) significantly impacts post‐kidney transplantation (KTx), leading to delayed graft function (DGF) and potential graft loss. Current biomarkers and therapies for DGF and graft survival are inadequate. Immune cell infiltration after renal IRI is crucial in driving inflammation and injury.

**Methods:**

To address this, this study utilized microarray and RNA‐seq datasets from the Gene Expression Omnibus (GEO) database to identify differentially expressed immune infiltration‐related genes (DE‐IRGs) in IRI patients. Machine learning (ML) algorithms pinpointed hub DE‐IRGs, aiding in predictive model development and classification of post‐KTx IRI samples into clusters and risk groups. Regulatory networks incorporating transcription factors (TFs) and microRNAs (miRNAs) were constructed using NetworkAnalyst 3.0, and predicted compounds/commonly used immunosuppressants were explored via Enrichr and molecular docking simulations.

**Results:**

Analysis revealed 47 DE‐IRGs, with hub genes (adrenomedullin [ADM], Serpin Family H Member 1 [SERPINH1], Solute carrier family 2 member 3 [SLC2A3], BCL‐2‐associated athanogene 3 [BAG3], NFKB inhibitor alpha [NFKBIA], Kruppel‐like factor 6 [KLF6], and CCAAT/enhancer‐binding protein delta [CEBPD]) linked to DGF and, in part, graft survival. Predictive models showed robust performance based on internal validation, with the DGF models achieving AUCs of 0.832 and 0.975 and graft survival models showing AUCs of 0.773, 0.742, and 0.757 for 1, 2, and 3 years, respectively. Higher immune cell infiltration correlated with adverse outcomes in cluster A or high‐risk groups. Key immune cells associated with DGF included activated CD8 T cells, activated dendritic cells (DCs), and effector memory CD4 T cells. Core regulatory TFs and miRNAs were identified, along with four core predicted compounds: acetaminophen, estradiol, valproic acid, and berbamine (which require further pharmacological validation), and three common immunosuppressants: cyclosporin A, mycophenolate mofetil (MMF), and tacrolimus.

**Conclusions:**

Our study identified potential hub genes most associated with immune cells during the post‐KTx IRI process, shedding light on the intricate interplay between genes, immune cells, and KTx outcomes.

## 1. Introduction

Kidney transplantation (KTx) is widely recognized as the optimal therapeutic strategy for patients with end‐stage kidney disease (ESKD) [1]. Compared to maintenance dialysis, KTx offers superior long‐term survival rates, enhanced quality of life, and greater cost‐effectiveness. However, the global burden of ESKD is escalating, creating a widening disparity between the demand for organs and the limited supply of donor kidneys [2–4]. To address this shortage, transplant centers have increasingly relied on expanded donors, such as donation after circulatory death (DCD) donors. While this strategy expands the donor pool, it is inevitably associated with a higher incidence of posttransplant complications, most notably delayed graft function (DGF) [5–7].

DGF, typically defined as the requirement for dialysis within the first week posttransplantation, represents a severe form of acute kidney injury (AKI) [6]. Its incidence is heavily influenced by donor characteristics, ranging from 3.1% to 5.1% in living donor recipients to as high as 29.0%–55.7% in recipients of deceased donor kidneys [7–10]. The clinical ramifications of DGF are profound; it prolongs hospitalization, complicates postoperative management, and significantly increases healthcare costs. More critically, DGF acts as a strong independent risk factor for acute rejection and is associated with reduced long‐term graft survival and increased patient mortality [11–14]. Despite these severe consequences, current clinical prediction tools—which rely largely on donor demographics such as age and cold ischemia time—often lack the sensitivity and specificity required for individual risk assessment [15, 16]. Consequently, there is an urgent need to identify robust molecular biomarkers that can accurately predict DGF and guide personalized therapeutic interventions.

The primary pathophysiological driver of DGF is ischemia‐reperfusion injury (IRI), an unavoidable event during organ retrieval, preservation, and implantation [17, 18]. IRI initiates a complex cascade beginning with hypoxic stress and endothelial dysfunction. Upon reperfusion, tissue injury is exacerbated by the generation of reactive oxygen species (ROS) and the rapid activation of inflammatory pathways [19, 20]. A hallmark of this process is the massive infiltration of immune cells into the graft. This infiltration is triggered by the release of damage‐associated molecular patterns (DAMPs)—such as high‐mobility group box 1 (HMGB1) and heat shock proteins—from necrotic tubular cells. These DAMPs interact with Toll‐like receptors (TLRs) on resident renal cells, recruiting innate immune cells, including neutrophils, macrophages, and dendritic cells (DCs), to the site of injury [21]. These infiltrating cells not only perpetuate tissue damage via the secretion of proinflammatory cytokines but also serve as a critical bridge to adaptive immunity. Activated antigen‐presenting cells migrate to lymphoid tissues to prime naïve T cells, leading to the subsequent infiltration of CD4+ and CD8+ T cells that can drive acute allograft rejection [22, 23]. Although animal studies suggest that targeting these immune pathways can alleviate renal injury [24–27], the specific transcriptomic signatures governing immune infiltration in human KTx patients remain insufficiently characterized.

Currently, therapeutic options to specifically prevent or treat DGF are limited, and no specific pharmacological agents have been approved for this indication [28–35]. The lack of reliable early biomarkers hinders the development of targeted therapies. Traditional experimental approaches often fail to capture the systemic complexity of the immune response. In contrast, integrative bioinformatics combined with machine learning (ML) offers a powerful, data‐driven approach to decipher high‐dimensional transcriptomic data. By leveraging large‐scale public datasets, ML algorithms can identify nonlinear relationships and pinpoint robust feature genes that traditional statistical methods might overlook [36–38]. Recent ML‐based studies have identified necroinflammation‐related biomarkers for DGF and renal allograft failure; however, immune infiltration‐related transcriptomic signatures in post‐KTx IRI remain poorly defined [39].

In this study, we hypothesized that the expression patterns of immune infiltration‐related genes (IRGs) in post‐IRI renal biopsies hold the key to predicting DGF and long‐term graft survival. To test this, we integrated multiplatform transcriptomic datasets (microarray and RNA‐seq) from the Gene Expression Omnibus (GEO) database. We employed a suite of advanced ML algorithms—including random forest (RF), support vector machine‐recursive feature elimination (SVM‐RFE), and least absolute shrinkage and selection operator (LASSO) regression—to screen for robust hub differentially expressed IRGs (DE‐IRGs). Utilizing these molecular signatures, we constructed predictive models for DGF and graft survival, stratified patients into distinct immunological risk groups, and elucidated the underlying regulatory networks involving transcription factors (TFs) and microRNAs (miRNAs). Furthermore, to facilitate translational potential, we performed in silico molecular docking to predict small‐molecule compounds that could potentially modulate these hub targets. This study aims to provide a comprehensive landscape of the immune‐molecular mechanisms driving DGF and to offer novel, robust biomarkers and therapeutic candidates for improving kidney transplant outcomes.

## 2. Materials and Methods

### 2.1. Dataset Processing

The investigation commenced with the pivotal step of expression profiling, employing either array or high‐throughput sequencing methodologies. A meticulous selection process led to the identification of three datasets (Supporting Information 1: Table S1). The GSE43974 dataset presents a comprehensive collection of 188 prerecovery and 203 postischemia/reperfusion (IR) renal biopsies [40]; the GSE90861 dataset comprises 23 pairs of pre‐ and postreperfusion biopsies, which included of 11 immediate graft function (IGF) samples and 12 DGF samples [41]; the GSE21374 dataset encompasses 282 late post‐KTx biopsies, among which 206 have demonstrated survival durations of no more than 3 years [42]. Using the matching platform files, gene symbols corresponding to each probe matrix were obtained. For the GSE43974 microarray dataset, the “neqc” function from the “limma” package was employed for background correction and normalization. In the case of the GSE90861 RNA‐seq dataset, variance‐stabilizing transformation was performed using the DESeq2 package with the “vst” function. For the GSE21374 microarray dataset, normalization was conducted using the “limma” package with the “normalizeBetweenArrays” function. All data analyses were carried out using R (Version 4.2.3).

### 2.2. Collection of IRGs

A comprehensive exploration into the realm of immune infiltration led to the acquisition of 2903 IRGs (Supporting Information 1: Table S2). This was achieved through meticulous searches conducted on GeneCards (https://www.genecards.org/), employing stringent criteria such as category protein‐coding and relevance scores, with selection based on those ranking in the upper half.

### 2.3. DE‑IRGs Identification and External Validation

In the analysis of the GSE43974 dataset, the R package “limma” was utilized to discern the positive DEGs, employing a false discovery rate (FDR) < 0.05 and a log_2_ fold change (logFC) > 1. The identification of DE‐IRGs was accomplished by intersecting the DEGs with IRGs, as illustrated by the Venn network generated through EVenn [43]. Furthermore, the GSE90861 dataset was employed as an independent cohort to validate the expression patterns of DE‐IRGs after the IRI.

### 2.4. Exploration of the Protein–Protein Interaction (PPI) Network of DE‑IRGs

The PPI network elucidates established and prospective associations among the proteins encoded by the 47 DE‐IRGs. This network was meticulously constructed utilizing the STRING database [44], adhering to specific parameters (organism: *Homo sapiens*; full STRING network; medium confidence: 0.400; FDR stringency < 0.05).

### 2.5. Enrichment Analysis

The Gene Ontology (GO) and Kyoto Encyclopedia of Genes and Genomes (KEGG) enrichment analyses of DE‐IRGs were performed utilizing the R package “clusterProfiler” [45]. Gene annotations from “org.Hs.eg.db” [46] and the KEGG REST API (https://www.kegg.jp/kegg/rest/keggapi.html) [47] served as the background datasets. Significantly enriched functions and pathways were identified based on an FDR threshold of < 0.05.

### 2.6. Analysis of Immune Cell Infiltration

To elucidate the immune cell infiltration associated with DE‐IRGs and their respective subgroups, we employed a robust R package, IOBR [48]. This package amalgamates various existing microenvironmental deconvolution methodologies and signature construction tools. Single‐sample gene set enrichment analysis (ssGSEA) was leveraged to estimate the abundance of immune cell populations and assess their activity within each sample. The abundance of innate immune cells, including macrophages and neutrophils, in the DGF subtypes was further corroborated using quanTIseq, xCELL, and MCP‐counter methodologies. Additionally, correlation analysis between immune cell infiltration and DE‐IRGs was performed using the R function “corr.test” from the “psych” package.

### 2.7. Identification of DGF‐Related Hub DE‐IRGs by ML Methods

We employed three distinct ML methods to screen for DGF‐related hub DE‐IRGs. The RF algorithm [49], a supervised classification method utilizing an ensemble of decision trees, was applied via the “randomForest” R package. Features were ranked based on the Gini importance measure, and in our study, we selected the top 10 ranked genes. The SVM‐RFE algorithm, implemented through the “caret” R package, is a recursive feature elimination strategy. It utilizes weighted vectors generated from the SVM to optimize classification accuracy across different groups. By intersecting the top‐ranked genes identified by the RF algorithm with those obtained through the SVM‐RFE algorithm, we ultimately delineated RF/SVM‐RFE feature DE‐IRGs.

Leveraging the background of RF/SVM‐RFE feature DE‐IRGs, 202 post‐IRI early KTx samples from the GSE43974 dataset, characterized by graft function, were stratified into training and testing sets at a 7:3 ratio using the R package “caret.” In the training set, tenfold cross‐validated LASSO regression was conducted, facilitated by the R package “glmnet.” The selection of key λ values was based on binomial deviance, utilizing both the minimum criterion (lambda.min) and the 1 standard error of the minimum criterion (lambda.1se). Optimal λ, as determined through the minimum criterion, guided the identification of hub genes and their corresponding coefficients for subsequent model construction. Subsequently, DGF‐related hub DE‐IRGs were identified along with their respective regression coefficients.

### 2.8. Consensus Clustering

To delineate distinct subtypes of immune infiltration samples, a cluster analysis of all IRI samples within the GSE43974 dataset was conducted. This analysis relied on the expression patterns of DGF‐related hub DE‐IRGs and was executed using the R package “ConsensusClusterPlus” [5]. The number of clusters (*k*) was varied from 2 to 8, employing the “partitioning around medoids” cluster algorithm with the “Spearman” distance metric. Moreover, 80% of the samples were subjected to resampling for 500 repetitions. The determination of the optimal *k* value was predicated on the best item‐consensus (IC) and cluster‐consensus (CLC) performances.

### 2.9. ML‑Driven Construction of the Predictive Models for Post‑KTx DGF

By employing a linear combination of previously acquired regression coefficients from the DGF training set and the expression levels of DGF‐related hub DE‐IRGs, risk scores were computed (Riskscore = ∑*n i* = 1[geneCoef *i* × geneExpi]), facilitating model construction. The performance of the model was assessed through receiver operating characteristic (ROC) curves based on these risk scores, examined across the training, testing, and all sets. Additionally, the model’s performance was assessed by calibration curve analysis, alongside the Hosmer–Lemeshow test, to evaluate the concordance between predicted and actual probabilities by using the R packages “rms” and “ResourceSelection.” Furthermore, risk scores were utilized to categorize IRI patients into high‐ and low‐risk groups for subsequent analyses, employing a middle‐ranking cutoff. To strengthen the clinical relevance of our predictive model, we further developed a hub DE‐IRG‐based clinical model for DGF prediction by incorporating the sole clinical variable available from the dataset: donor type. Using the RF algorithm, the model was constructed with a 7:3 split into training and testing sets. Its performance was also evaluated via ROC curves and calibration curve analysis, including the Hosmer–Lemeshow test.

### 2.10. Identification and Correlation Analysis of Core Infiltrated Immune Cells With DGF‐Related Hub DE‐IRGs

Following the assessment of immune activity in each sample by ssGSEA, we proceeded to identify differentially infiltrated immune cells (DI‐ICs) between pre‐ and post‐IR. Subsequently, corresponding samples from the GSE43974 dataset were selected based on the DI‐ICs. Utilizing the LASSO regression algorithm with tenfold cross‐validation, we identified core DI‐ICs. Subsequently, their correlation with DGF‐related hub DE‐IRGs was examined using the R function “corr.test” from the “psych” package.

### 2.11. Construction of the DGF‐Related Hub DE‐IRGs Regulatory Networks

To delve deeper into the transcriptional regulatory mechanisms governing DGF‐related hub DE‐IRGs, we sought to elucidate the involvement of regulatory TFs and miRNAs. Utilizing NetworkAnalyst 3.0 (https://www.networkanalyst.ca/) [50], a web‐based platform renowned for processing, functionally analyzing, and visualizing various forms of gene expression data, we aimed to identify TFs and miRNAs interacting with DGF‐related hub DE‐IRGs. Subsequently, we constructed TF‐hub DE‐IRGs and TF‐miRNA‐hub DE‐IRG regulatory networks.

### 2.12. Prediction of Compounds Targeting DGF‐Related Hub DE‐IRGs

Following the identification of DGF‐related hub DE‐IRGs, our subsequent endeavor involved screening for emerging compounds capable of targeting these hub genes. Leveraging the online platforms Enrichr and the Drug Signature Database (DSigDB), we embarked on the prediction of drug molecules. Enrichr (https://maayanlab.cloud/Enrichr/) hosts a comprehensive collection of ~400,000 annotated gene sets organized into 300 gene‐set libraries, facilitating detailed and reliable analysis [51, 52]. Meanwhile, DSigDB comprises a repository of drug‐ and small‐molecule‐related gene sets derived from quantitative inhibition and/or drug‐induced gene expression change data [53]. Access to DSigDB was facilitated through Enrichr under the Diseases/Drugs function. The predicted compounds and molecules were comprehensively ranked based on their odds ratio (adjusted *p*‐value < 0.05). Following a meticulous evaluation, the top 15 predicted compounds were selected based on their potential immunosuppressive effects for further analysis. Furthermore, four compounds were chosen for subsequent molecular docking simulations and identification based on their top‐ranking positions and their potential targeting genes.

### 2.13. Molecular Docking Simulation and Identification for Predicted Compounds and Common Immunosuppressants

The molecular docking approach was employed to identify the interactions between the predicted compounds, common immunosuppressants, and the DGF‐related hub DE‐IRGs, which is commonly utilized in drug discovery as it enables predictions of novel binding modes, predictions of ligand‐target interactions at a molecular level, and delineation of structure–activity relationships [54]. It should be noted that cyclosporin A, mycophenolate mofetil (MMF), and tacrolimus were included in this phase of analysis, as they are among the most commonly used immunosuppressants in the field of renal transplantation. The crystal protein structures of the hub DE‐IRGs were obtained from the RCSB Protein Data Bank (https://www.rcsb.org/) and the AlphaFold Protein Structure Database (https://alphafold.ebi.ac.uk/) [55, 56]. The structure files of the predicted compounds in the “SDF” format were downloaded via the ZINC (https://zinc.docking.org/) or PubChem databases (https://pubchem.ncbi.nlm.nih.gov/). Protein conformations were modified using PyMOL 2.5.8 and AutoDockTools 1.5.7, including the removal of the existing ligands and water, addition of hydrogen, optimization of amino acids, and calculation of charges. The predicted compounds served as the ligands and the hub DE‐IRGs’ proteins as the receptors. The binding conformation between the ligand and receptor was predicted by AutoDock Vina, known for improving the average accuracy of the binding mode predictions compared to AutoDock 4 [57, 58]. The binding energy, a weighted average of the docking score to assess the reliability, was used to assess the potential of the ligand‐receptor binding affinity. Binding energy greater than −4 kcal/mol indicates a relatively weak binding interaction between the ligand and receptor. Binding energies in the range of −4 to −7 kcal/mol suggest a moderate binding interaction between the ligand and the receptor. Conversely, binding energies of less than −7 kcal/mol indicate a strong binding affinity between the ligand and receptor. Finally, the visualization of molecular interactions between the proteins and ligands was performed using PyMOL 2.5.8.

### 2.14. Construction of the Allograft Survival Predictive Model

Through univariable Cox regression analysis (hazard ratio ≠ 1 and *p*‐value < 0.05), we screened for allograft survival‐related RF/SVM‐RFE feature IRGs, utilizing the R package “survival,” and referencing the GSE21374 dataset.

From the GSE21374 dataset, survival‐related DE‐IRG samples were extracted and then randomly divided 7:3 into training and testing sets. Employing the LASSO regression algorithm with tenfold cross‐validation in the training set, allograft survival‐related hub DE‐IRGs and their coefficients were established. Risk scores (Riskscore = ∑*n i* = 1[geneCoef *i* × geneExpi]) were instrumental in facilitating model construction. The performance of the model was illustrated using time‐dependent ROC curves and time‐dependent calibration curves, the latter accompanied by the Hosmer–Lemeshow test. Based on the risk scores and their median, 206 samples with graft status information within 3 years (155 graft survival and 51 graft loss samples; 56 rejection and 150 nonrejection samples) were subsequently categorized into two different risk groups for graft loss. Prognostic differences between the two groups were assessed using Kaplan–Meier graft survival analysis. Cox proportional hazards (PHs) regression analysis was employed to evaluate the different prognostic hazards between the two groups. The Schoenfeld residuals test was utilized to determine whether the PH assumption was violated based on the probability of the correlation statistics. These analyses were conducted using R packages “survival,” “survminer,” “caret,” “rms,” “ResourceSelection,” and “glmnet.”

### 2.15. Identification of the Clinical Significance of Hub DE‐IRG Targets

To investigate the potential molecular determinants associated with renal IRI and DGF, we identified seven core hub DE‐IRGs. The clinical relevance of these genes (including adrenomedullin [ADM], Serpin Family H Member 1 [SERPINH1], Solute carrier family 2 member 3 [SLC2A3], BCL‐2‐associated athanogene 3 [BAG3], NFKB inhibitor alpha [NFKBIA], Kruppel‐like factor 6 [KLF6], and CCAAT/enhancer‐binding protein delta [CEBPD]) was further validated using the Nephroseq v5 database (http://v5.nephroseq.org/), a comprehensive resource for renal transcriptomic profiling. Specifically, we assessed the correlations between the expression of these hub genes and kidney function parameters in transplant recipients, focusing on the glomerular filtration rate (GFR) estimated by the Modification of Diet in Renal Disease (MDRD) or Chronic Kidney Disease Epidemiology Collaboration (CKD‐EPI) equations.

### 2.16. Animals, UIRI Model, and Real‐Time Quantitative PCR (RT‐qPCR) Analysis

Male C57BL/6N mice (6–8 weeks old) were purchased from Beijing Vital River Laboratory Animal Technology Co., Ltd. (Beijing, China) and housed in a specific pathogen‐free (SPF) environment. The mice were randomly assigned to the Sham group (*n* = 7) and the UIRI group (*n* = 7). All mice were fasted for 12 h prior to surgery. Anesthesia was induced with 3% isoflurane and maintained at 1% during the procedure, with core body temperature strictly maintained at 37°C using a heating pad. To establish the unilateral renal IRI (UIRI) model, the right kidney was excised, and blood flow to the left kidney was occluded at the renal pedicle using a nontraumatic sterile vascular clamp for 20 min. Subsequently, the clamp was removed to initiate reperfusion. Normal saline (0.5 mL) was administered intraperitoneally to prevent dehydration. Sham‐operated control mice underwent right nephrectomy and exposure of the left renal pedicle without clamping. All mice were euthanized 24 h postoperation, and kidney tissues alongside serum samples were collected for subsequent analyses.

For histological assessment, the renal tissues were fixed in 4% paraformaldehyde, dehydrated, and embedded in paraffin. The embedded tissues were then sectioned, deparaffinized, and rehydrated. Hematoxylin and eosin (H&E) staining was performed to observe morphological changes in the kidney.

To evaluate in vivo gene expression, RT‐qPCR was performed. Total RNA was isolated from the IRI kidney tissues using the SteadyPure Universal RNA Extraction Kit (Accurate Biotechnology Co., Ltd., Hunan, China) according to the manufacturer’s instructions. The extracted RNA was reverse‐transcribed into cDNA utilizing the Evo M‐MLV RT Mix Kit (Accurate Biotechnology Co., Ltd., Hunan, China). PCR was conducted on a Real‐Time PCR Detection System (Applied Biosystems, USA) with the SYBR Green Pro Taq HS qPCR Kit (Accurate Biotechnology Co., Ltd., Hunan, China). Relative gene expression was normalized to GAPDH levels and calculated using the 2^-ΔΔCt^ method. Primer sequences used in this study are detailed in Supporting Information 1: Table S9. All analyzed samples represented independent biological replicates. Comparisons between the Sham and UIRI groups were performed using the nonparametric Mann–Whitney *U* test. All surgical procedures, animal handling, and husbandry were performed in accordance with the guidelines of the Institutional Animal Care and Use/Ethics Committee of Beijing Chaoyang Hospital (Ethics Number 2023‐animal‐97).

### 2.17. Statistical Analysis

Statistical analysis was performed using R software (Version 4.2.3). Descriptive statistics were utilized to characterize the distributions of continuous and nominal variables. Student’s *t*‐test and Mann–Whitney *U* nonparametric test were employed for variables with normal and abnormal distributions, respectively. Differences between the two IRI subtypes and risk groups of classified data were assessed using a two‐sided Chi‐square test, with Fisher’s exact test utilized when the total sample size was less than 40. Spearman’s correlation method was utilized to determine the association between genes, cells, and immune characteristics. Statistical significance was defined as a *p*‐value or FDR < 0.05.

## 3. Results

### 3.1. The Expression Abundance and Identification of DE‐IRGs in KTx Patients

**Figure 1:**
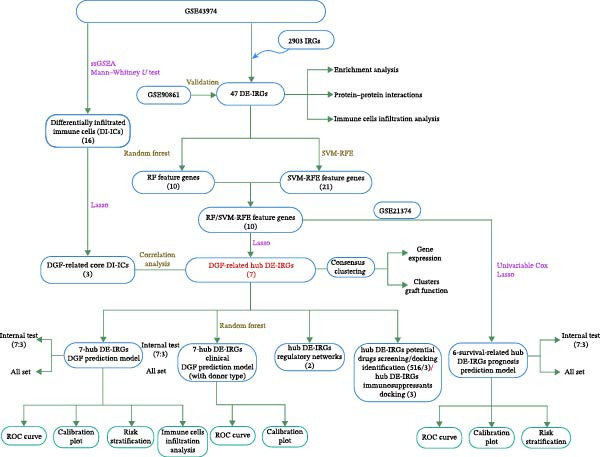
Flowsheet of this study. DE‐IRGs, differentially expressed immune infiltration‐related genes; DGF, delayed graft function; DI‐ICs, differentially infiltrated immune cells; IRGs, immune infiltration‐related genes; LASSO, least absolute shrinkage and selection operator; RF, random forest; ROC, receiver operating characteristic; ssGSEA, single‐sample gene set enrichment analysis; SVM‐RFE, support vector machine recursive feature elimination.

Figure 1 demonstrates the flowsheet of this study. A total of 132 upregulated DEGs were revealed after a comprehensive differential analysis of gene expression profiles from 188 pre‐IR and 203 post‐IR kidney biopsy samples, obtained after undergoing cold ischemia and recovering 1 h postreperfusion (Figure 2A). Considering the intricate relationship between the functionality of KTx patients’ allografts and immune infiltration processes, 2903 IRGs were obtained through thorough searches in GeneCards (Supporting Information 1: Table S2). Through the intersection of DEGs and IRGs, 47 upregulated genes (Supporting Information 1: Table S3) were identified as DE‐IRGs, as depicted in the Venn network (Figure 2B). The expression landscape of these 47 DE‐IRGs in both control and post‐IR kidney biopsies was further elucidated through the heat map and box plot (Figure 2C,D). The genes highlighted in Figure 2A,C,D represent DGF‐related hub DE‐IRGs. To minimize any potential influence of chance and further determine the expression, the expression of these 47 DE‐IRGs was validated in an independent set of KTx profiles (GSE90861). The results consistently demonstrated the upregulation of these 47 genes, all of which exhibited significant differences, as depicted in both the raincloud plot and heat map (Figure 2E,F). This procedure identified 47 upregulated DE‐IRGs linked to DGF in KTx patients following cold ischemia and 1‐h postreperfusion. These genes were derived from the intersection of 132 DEGs and 2903 IRGs, and their expression was consistently validated in an independent dataset.

Figure 2Identification of the 47 DE‐IRGs and validation in post‐IRI KTx samples. (A) Volcano plot displaying a total of 132 upregulated DEGs. (B) Venn diagram illustrating the intersection of DEGs and IRGs. (C) Heatmap depicting the upregulated expression of the 47 DE‐IRGs in pre‐IR and post‐IR samples. (D) Box plot demonstrating the expression of the 47 DE‐IRGs pre‐ and post‐IR, with all showing statistical significance. (E) Raincloud plots revealing the upregulated expression of the 47 DE‐IRGs in the validation datasets GSE90861. (F) Heatmap illustrating the upregulated expression of the 47 DE‐IRGs in validation datasets GSE90861. DEGs, differentially expressed genes; IRGs, immune infiltration‐related genes; red‐labeled genes, seven DGF‐related hub DE‐IRGs; IR, ischemia‐reperfusion;  ^∗^ *p*  < 0.05;  ^∗∗^ *p*  < 0.01;  ^∗∗∗^ *p*  < 0.001.

**Figure:**
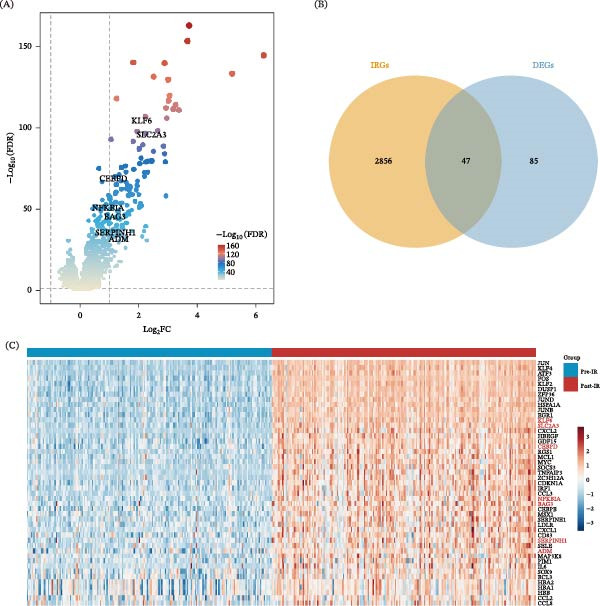

**Figure:**
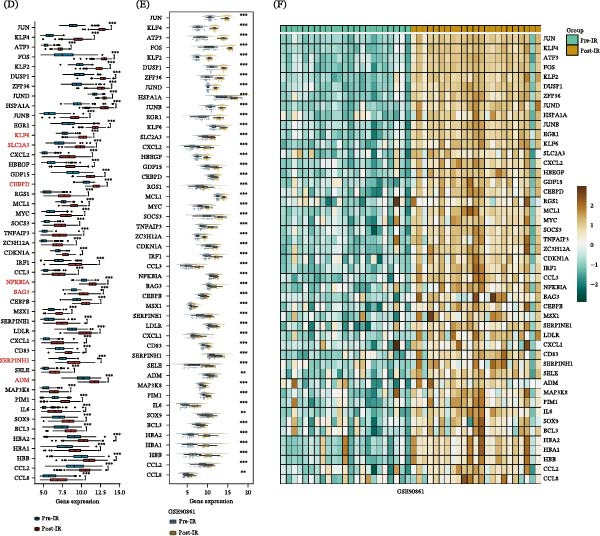

### 3.2. PPIs, Biological Functions, and Immune Correlations of DE‑IRGs

The 47 DE‐IRGs were subjected to functional enrichment analysis to unveil their potential biological associations and functions. The PPI network illustrated a high degree of interaction among the encoded proteins, with three proteins (HBB, HBA1, and HBA2) standing apart from other protein interaction networks (Figure 3A). This interaction identification spanned multidimensional channels, including curated databases, experimental determinations, text mining, coexpression, and protein homology. Subsequent GO analysis revealed the involvement of these genes in various biological, molecular, and cellular functions such as response to molecules of bacterial origin, response to interleukin‐1, chemokine activity, and chemokine receptor binding. These functions align with the concept of immune infiltration triggered by specific biological stimuli. Moreover, these findings are in line with the annotation results of KEGG analysis, which identified TNF, IL‐7, NOD‐like receptor, TLR signaling pathways, and other pathways associated with infectious or autoimmune diseases (Figure 3B,C).

Figure 3Protein–protein interactions, biological functions, and immune correlations of DE‑IRGs. (A) Protein–protein interactions among the 47 DE‐IRGs, revealing a strong relationship with each other. (B) GO analysis plot showing the enrichment of DE‐IRGs in processes responding to molecules of bacterial origin, interleukin‐1, chemokine activity, chemokine receptor binding, etc. (C) KEGG analysis plot showing enrichment of DE‐IRGs in TNF, IL‐17, NOD‐like receptor, and Toll‐like receptor signaling pathways, etc. (D) Immune cell infiltration analysis plot showing significant infiltration of up to 16 types of immune cells into the kidneys after IR. (E) Correlation heat map depicting robust associations between most of the 47 DE‐IRGs and DI‐ICs, with MAP3K8 exhibiting the strongest positive correlation with mast cells, while CEBPD displayed the most negative correlation with immature dendritic cells. DI‐ICs, differentially infiltrated immune cells; GO, Gene Ontology; KEGG, Kyoto Encyclopedia of Genes and Genomes; ns, not significant;  ^∗^ *p*  < 0.05;  ^∗∗^ *p*  < 0.01;  ^∗∗∗^ *p*  < 0.001.

**Figure:**
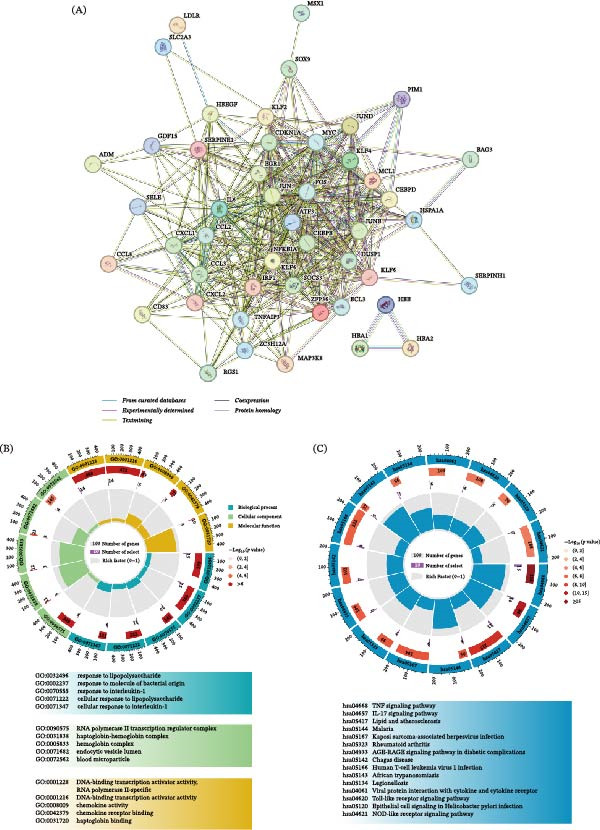

**Figure:**
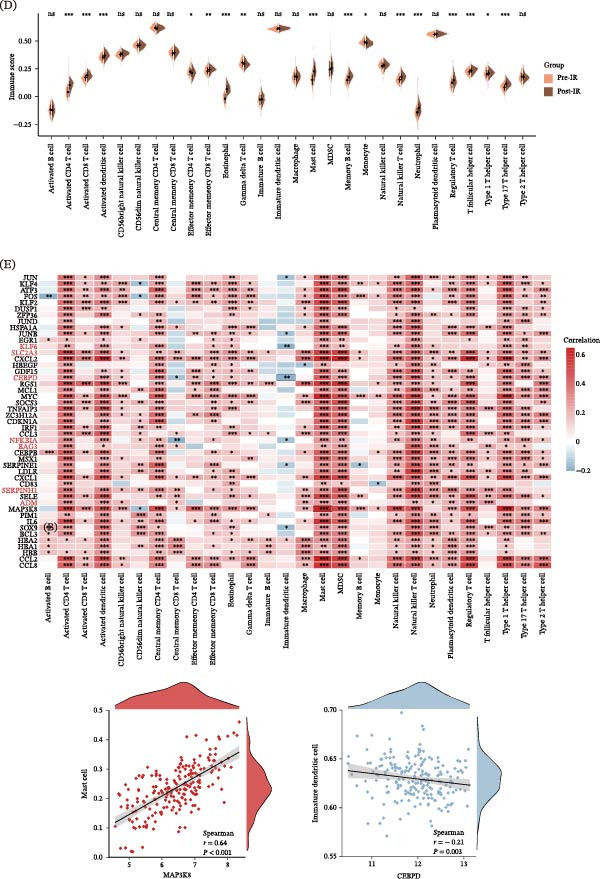

Given that the primary function of DE‐IRGs revolves around immune infiltration, our investigation delved into the immune characteristics associated with these genes. Initially, we assessed the differences in immune cell abundance during the early stages of post‐IR compared to pre‐IR. Our immune cell infiltration analysis revealed the presence of up to 16 types of immune cells significantly infiltrating the kidneys after IR, including innate immune cells such as DCs, granulocytes, mast cells, monocyte macrophages, and various adaptive immune cells (Figure 3D). Subsequently, the correlation heat map illustrated robust associations between most of the 47 DE‐IRGs and these DI‐ICs, primarily displaying positive correlations, thereby underscoring the pivotal significance of DE‐IRGs in immune infiltration processes (Figure 3E). Notably, we highlighted the most positive and negative associations: MAP3K8 exhibited the strongest positive correlation with mast cells (*r* = 0.64, *p*  < 0.001), while CEBPD displayed the most negative correlation with immature DCs (*r* = −0.21, *p* = 0.003; Figure 3E). The functional enrichment and PPI analysis of the 47 DE‐IRGs revealed their close involvement with each other and immune processes, such as chemokine activity and response to interleukin‐1, with key pathways including TNF and TLR signaling. Immune cell infiltration analysis showed 16 types of immune cells significantly increased after IR, including both innate and adaptive immune cells. MAP3K8 had a strong positive correlation with mast cells, while CEBPD was negatively correlated with immature DCs, highlighting the role of DE‐IRGs in driving immune responses after kidney injury.

### 3.3. Identification of DGF‐Related Hub DE‐IRGs and Construction of DGF Predictive Models

**Figure 4:**
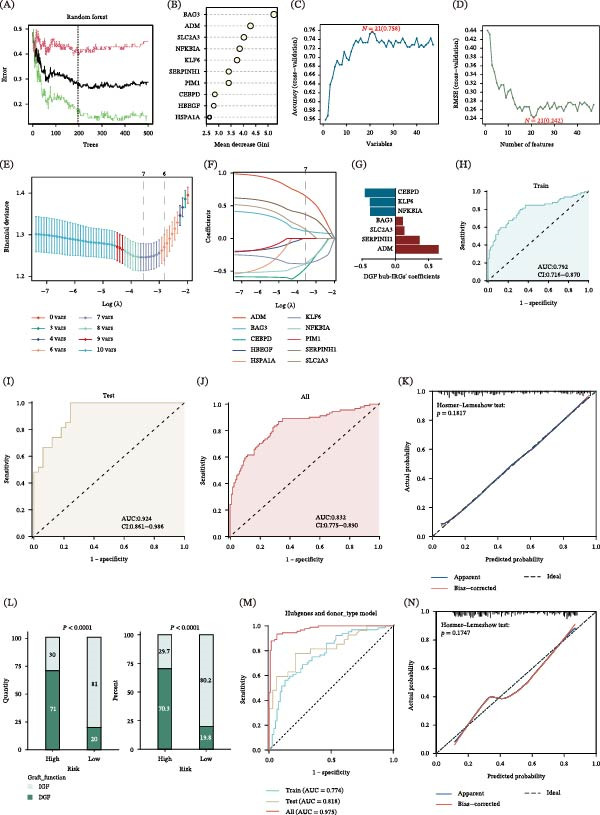
Identification of DGF‐related hub DE‐IRGs and construction of the DGF predictive models. (A) Random forest tree illustrating the separation of non‐DGF samples (red line), DGF samples (green line), and overall samples (black line), with the dotted line representing the tree with the minimum error rate. (B) Gini importance plot ranking the top 10 DGF‐related RF feature IRGs based on mean decrease Gini from the RF algorithm, all of which were included in the 21 DGF‐related SVM‐RFE feature genes. (C–D) Plots of SVM‐RFE accuracy (C) and RMSE (D) demonstrating optimal predictive performance when the variable genes were selected as 21, identified through tenfold cross‐validation. (E) Determination of optimal hub genes as seven, indicated by the minimum binomial deviance, with vertical dotted lines denoting lambda.min (left) and lambda.1se (right) in tenfold cross‐validation. (F) LASSO coefficient profiles of the 10 RF/SVM‐RFE feature DE‐IRGs, with the dotted line drawn at the optimal λ resulting in seven coefficients. (G) Coefficients of the hub DE‐IRGs. (H–J) ROC curves illustrating the performance of the DGF predictive model on the training set (H), internal test set (I), and all sets (J), with AUC values of 0.792, 0.924, and 0.832, respectively. (K) Calibration curve plot of DGF predictive model showing close alignment between the apparent and bias‐corrected curves with the ideal curve. (L) Stacked bar charts indicating a higher occurrence of DGF in the high‐risk group. (M) ROC curves illustrating the performance of the clinical DGF predictive model in the training, internal testing, and overall sets, with AUC values of 0.774, 0.818, and 0.975, respectively. The AUC of 0.975 for the “all set” was calculated on the combined training and testing samples and does not represent external validation. (N) Calibration curve plot of DGF clinical predictive model showing close alignment between the apparent and Bias‐corrected curves with the ideal curve. AUC, area under the curve; CI, confidence interval; RF, random forest; RMSE, root mean square error; SVM‐RFE, support vector machine recursive feature elimination.

Through the RF algorithm, we identified DGF‐related RF feature IRGs, ranking the top 10 according to the Gini importance measure (Figure 4A,B). Concurrently, we employed the SVM‐RFE algorithm to screen DGF‐related SVM‐RFE feature genes, determining the number of genes based on the highest accuracy (lowest error) (Figure 4C,D). Through the intersection of the top 10 feature genes identified by the RF algorithm and the 21 feature genes screened by the SVM‐RFE algorithm, we ultimately identified 10 RF/SVM‐RFE feature DE‐IRGs, with all the top 10 RF feature genes included in the 21 SVM‐RFE feature genes (Figure 4B).

To further detect the DGF‐related hub DE‐IRGs, we utilized the LASSO regression algorithm within the training set, comprising 70% of the 202 IRI samples randomly selected from the GSE43974 dataset. This analysis was based on the expression profiles of the 10 RF/SVM‐RFE‐featured DE‐IRGs. Optimal hub genes were determined to be seven, as indicated by the minimum Binomial Deviance (Figure 4E). Subsequently, the seven identified DGF‐related hub DE‐IRGs (CEBPD, KLF6, NFKBIA, BAG3, SLC2A3, SERPINH1, and ADM) were selected for constructing the DGF predictive model, along with their corresponding regression coefficients (−0.458, −0.385, −0.381, 0.112, 0.137, 0.366, and 0.650, respectively) (Figure 4E–G; Supporting Information 1: Table S4). Following model construction, risk scores were computed for each IRI sample (Riskscore = −0.458 × CEBPD expression − 0.385 × KLF6 expression −0.381 × NFKBIA expression + 0.112 × BAG3 expression + 0.137 × SLC2A3 expression + 0.366 × SERPINH1 expression + 0.650 × ADM expression), and all samples were stratified into high‐risk and low‐risk groups based on the median risk score. The performance of the model was evaluated using the ROC curve, with the AUC of risk scores calculated for the training set, internal testing set, and overall set, yielding values of 0.792, 0.924, and 0.832, respectively (Figure 4H–J). Additionally, the calibration curve analysis plot demonstrated close alignment between the apparent and bias‐corrected curves with the ideal curve, indicating that the predicted probabilities from our model closely match the actual probabilities. This finding was further supported by the Hosmer–Lemeshow test, which yielded a *p*‐value of 0.1817, indicating no statistical difference between the predicted and actual probabilities (Figure 4K). The IRI samples were divided based on their risk scores: high‐risk group (*n* = 101), low‐risk group (*n* = 101), revealing a notable disparity between the high‐risk and low‐risk groups in terms of DGF occurrence (71 vs. 20, 70.3% vs. 19.8%, *p*  < 0.0001; Figure 4L). To further enhance the model’s relevance for clinical practice, we next developed a clinical predictive model for DGF based on the seven hub DE‐IRGs and a clinical variable: donor type (living, DBD, and DCD). This model was built using the RF algorithm, and 70% of the 202 IRI samples were randomly selected from the GSE43974 dataset. Model performance was assessed using ROC curves, with AUC values calculated for the training set, internal testing set, and overall set, yielding promising AUCs of 0.774, 0.818, and 0.975, respectively (Figure 4M). Notably, the model demonstrated strong performance in the overall set, with an AUC close to 1. Calibration curve analysis showed strong agreement between the apparent and bias‐corrected curves and the ideal curve, further supported by the Hosmer–Lemeshow test, which produced a *p*‐value of 0.1747 (Figure 4N). Leveraging ML algorithms, this procedure identified seven hub DE‐IRGs (CEBPD, KLF6, NFKBIA, BAG3, SLC2A3, SERPINH1, and ADM) related to DGF and constructed two predictive models. The original model effectively stratified kidney IRI samples into high‐ and low‐risk groups, with a significant difference in DGF occurrence between these groups. Incorporating donor type as a clinical variable, the clinical predictive model showed strong performance with high AUC values across training, testing, and overall datasets, indicating its potential utility in clinical settings for predicting DGF risk.

### 3.4. Stratification of Hub DE‐IRGs Related Subtypes in the IRI Patients

**Figure 5:**
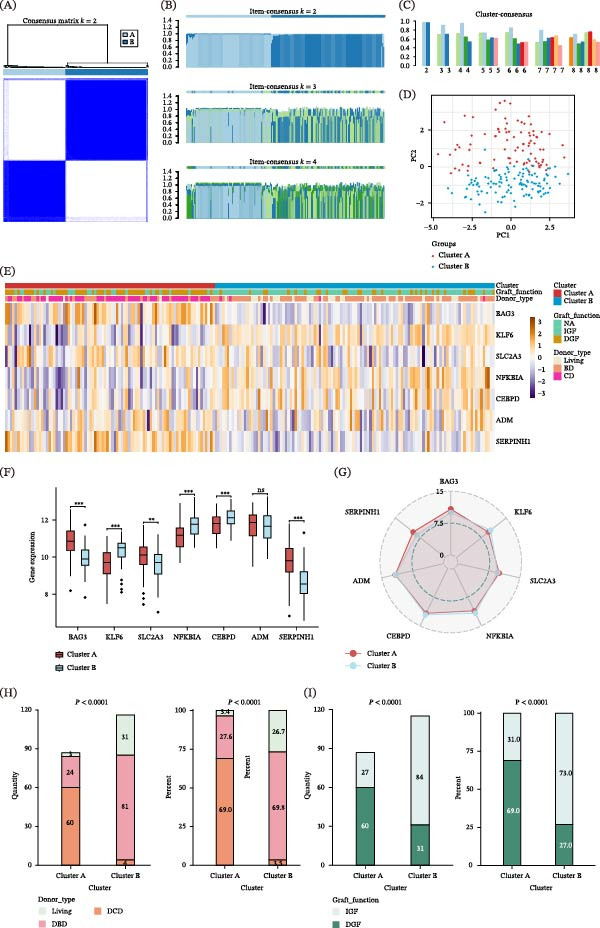
Stratification of hub DE‐IRG‐related subtypes in the IRI patients. (A) Consensus clustering matrix for *k* = 2. (B) Item‐consensus plot (*k* = 2–4), with *k* = 2 showing the optimal performance. (C) Cluster‐consensus plot displaying different CLC values (*k* = 2–8), with *k* = 2 exhibiting the highest stability. (D) PCA plot showing expression patterns of hub DE‐IRGs between the two clusters. (E–G) Heatmap (E), box plot (F), and radar plot (G) illustrating the expression differences of the seven hub DE‐IRGs between the two subtypes, with cluster A displaying higher expression levels of BAG3, SLC2A3, and SERPINH1, while KLF6, NFKBIA, and CEBPD are downregulated; ADM shows no differential expression between the two clusters. (H–I) Stacked bar charts indicating a higher occurrence of DCD donor sources (H) and DGF (I) in the cluster A. CLC, cluster‐consensus; DBD, brain‐dead; DCD, donation after circulatory death; DGF, delayed graft function; IGF, immediate graft function; ns, not significant;  ^∗^ *p*  < 0.05;  ^∗∗^ *p*  < 0.01;  ^∗∗∗^ *p*  < 0.001.

The degree of IRI manifests diversely among patients, leading to varying levels of immune infiltration in the kidney and subsequent tubular cell death, thereby influencing graft functions differently. Consequently, our study aimed to discern discrepancies between IRI subtypes by stratifying 203 IRI patients based on the expression patterns of the seven DGF‐related hub DE‐IRGs using a consensus clustering algorithm. We prioritized the IC and CLC performances to determine the optimal number of clusters (*k*). IC denotes the average consensus value between an item and members of a consensus cluster, facilitating the identification of highly representative samples and those with mixed cluster associations across different *k* values. Meanwhile, CLC represents the average pairwise IC of items in a consensus cluster, offering insights into the stability of existing clusters upon adding new ones [45]. As depicted in the IC plot, when *k* = 2, most samples were distinctly represented by unique clusters, whereas an increase in *k* led to mixed cluster associations among samples (Figure 5B). Meanwhile, the CLC plot indicated that when *k* = 2, subclusters exhibited the highest CLC value, signifying enhanced stability (Figure 5C). Ultimately, after comparing various k values (Supporting Information 2: Figure S1), we determined that the IRI samples should be effectively categorized into two subtypes (Figure 5A), cluster A (*n* = 87) and cluster B (*n* = 116). Principal component analysis (PCA) further underscored the marked differences in hub DE‐IRG expression between the two IRI subtypes (Figure 5D).

The expression landscapes of the seven hub DE‐IRGs between the two subtypes were depicted using a heatmap (Figure 5E). Notably, compared to cluster B, cluster A exhibited higher expression levels of BAG3, SLC2A3, and SERPINH1, while KLF6, NFKBIA, and CEBPD were downregulated, with ADM showing no differential expression between the two clusters (Figure 5F,G). This differential expression pattern of the hub DE‐IRGs suggests potential differences in the extent of immune infiltration between the two subtypes. For instance, elevated levels of BAG3, SLC2A3, and SERPINH1 may lead to increased immune cell infiltration and subsequent cell damage [59–61]. Conversely, the downregulation of NFKBIA, a classical inhibitor of the NFκB pathway, appears to exacerbate immune cell infiltration [62]. Furthermore, the analysis revealed that donors in cluster A were predominantly obtained from DCD patients (60, 69.0%), while cluster B comprised a substantial proportion of donors from DBD and living patients (81, 69.8% and 31, 26.7%, *p*  < 0.0001; Figure 5H). These findings suggest that cluster B patients may experience milder immune infiltration and exhibit distinct clinical features. Notably, the incidence of DGF was significantly higher in cluster A compared to cluster B (60 vs. 31, 69.0% vs. 27.0%, *p*  < 0.0001; Figure 5I). This procedure stratified 203 IRI patients into two distinct subtypes based on the expression patterns of seven hub DE‐IRGs and consensus clustering outcomes. Cluster A and cluster B exhibited significant differences in hub DE‐IRG expression, which suggests greater immune infiltration and associated tissue damage in cluster A. Additionally, cluster A predominantly included donors from DCD and had a higher incidence of DGF compared to cluster B, which mostly included DBD and living donors. This stratification underscores the diverse impact of immune infiltration hub DE‐IRG expression patterns in predicting graft function.

### 3.5. Correlation With Core DI‐ICs and DGF‐Related Hub DE‐IRGs in Terms of DGF

To unravel the intricate relationships between DGF‐related hub DE‐IRGs and infiltrated immune cells influencing DGF occurrence, we employed the LASSO regression algorithm within the GSE43974 post‐IR samples, focusing on 16 DI‐ICs, such as activated CD4 T cell, activated CD8 T cell, activated DC, effector memory CD4 T cell, effector memory CD8 T cell, eosinophil, gamma delta T cell, mast cell, memory B cell, monocyte, natural killer T cell, neutrophil, regulatory T cell, T follicular helper cell, type 1 T helper cell, and type 17 T helper cell (Figure 3D). The determination of the core infiltrated cells’ number revealed three core DI‐ICs, as indicated by the minimum binomial deviance from the LASSO algorithm (Figure 6A). Subsequently, the DGF‐related three core DI‐ICs, namely, activated CD8 T cell, activated DC, and effector memory CD4 T cell, were identified (Figure 6B). The correlation analysis heat map further illustrated strong associations between the seven hub DE‐IRGs and the 16 DI‐ICs, predominantly revealing positive correlations, thus highlighting the crucial role of the hub DE‐IRGs in immune infiltration processes. Specifically, all seven hub DE‐IRGs exhibited significant positive correlations with activated DC, while only SLC2A3 and SERPINH1 significantly correlated with activated CD8 T cell, and only CEBPD significantly correlated with effector memory CD4 T cell (Figure 6C). Of particular note were the most positive and negative associations between hub DE‐IRGs and DI‐ICs: SERPINH1 exhibited the strongest positive correlation with natural killer T cell (*r* = 0.52, *p*  < 0.001; Figure 6D), while BAG3 displayed the most negative correlation with effector memory CD4 T cell, although statistical significance was not achieved (*r* = −0.13, *p* = 0.059; Figure 6E). This procedure identified three core DI‐ICs—activated CD8 T cells, activated DCs, and effector memory CD4 T cells—through LASSO regression analysis in post‐IR samples. The analysis revealed strong positive correlations between these core DI‐ICs and seven hub DE‐IRGs. Specifically, all hub DE‐IRGs were significantly correlated with activated DCs. These findings highlight the complex interplay between specific genes and immune cell subsets in the development of DGF.

Figure 6Identification of core DI‐ICs and myeloid immune infiltration, along with clinical correlations of the two DGF risk groups. (A) LASSO algorithm determined the core DI‐ICs leading DGF, indicated by the minimum Binomial Deviance, with vertical dotted lines denoting lambda.min (left) and lambda.1se (right) in tenfold cross‐validation. (B) LASSO coefficients plot of the 16 DI‐ICs, with the dotted line drawn at the most significant λ, activated CD8 T cell, highlighting activated CD8 T cells, activated dendritic cells, and effector memory CD4 T cells. (C) Correlation heat map demonstrating robust associations between most of the hub DE‐IRGs and DI‐ICs, with all seven hub DE‐IRGs exhibiting significant positive correlations with activated dendritic cells, while only SLC2A3 and SERPINH1 significantly correlate with activated CD8 T cells, and only CEBPD significantly correlates with effector memory CD4 T cells. Red‐labeled immune cells are core DI‐ICs. (D) SERPINH1 exhibits the strongest positive correlation with natural killer T cells. (E) BAG3 displays the most negative correlation with effector memory CD4 T cells. (F–I) Myeloid immune cell infiltration plot shows the differential immune cell infiltration of the two DGF risk groups, assessed via ssGSEA (F), quanTIseq (G), xCELL (H), and MCP‐counter (I) algorithms. (J) Stacked bar charts showing a higher occurrence of CD donor sources and composition of cluster A in the high‐risk group. (K) Sankey diagram illustrating the relationships among the donor type, graft function, IRI clusters, and the risk of IRI samples. BD, brain dead; CD, cardiac dead; DGF, delayed graft function; IGF, immediate graft function; ns, not significant; ssGSEA, single‐sample gene set enrichment analysis.  ^∗^ *p*  < 0.05;  ^∗∗^ *p*  < 0.01;  ^∗∗∗^ *p*  < 0.001.

**Figure:**
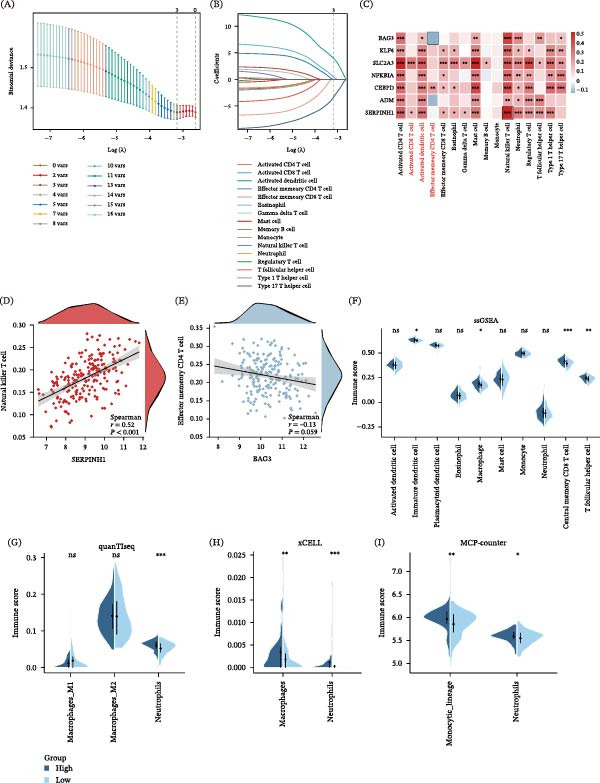

**Figure:**
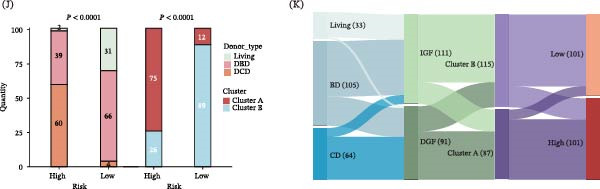

### 3.6. Myeloid Immune Infiltration and Clinical Characteristics of the Different DGF Risk Groups

Recent findings have highlighted the potential significance of myeloid immune cells, particularly macrophages and neutrophils, in contributing to tubular cell damage and kidney function [24–26, 63]. In light of this, we investigated the differences in myeloid immune cell infiltration between the two risk groups using ssGSEA. Our analysis unveiled a higher abundance of immature DCs and macrophages in the high‐risk group, while the abundance of neutrophils did not reach statistical significance. Notably, central memory CD8 T cells and T follicular helper cells were found to significantly infiltrate the high‐risk group (Figure 6F). Subsequently, we employed three additional algorithms—quanTIseq, xCELL, and MCP‐counter—to further assess the abundance of both macrophages and neutrophils. In contrast to ssGSEA, both xCELL and MCP‐counter algorithms demonstrated significant differences in the abundance of macrophages and neutrophils between the two groups. Conversely, the results from quanTIseq indicated that only neutrophils exhibited significant infiltration into the high‐risk group (Figure 6G–I).

The occurrence of DGF is intricately linked to the type of kidney donation, particularly with kidneys sourced from DCD donors, where a higher incidence of DGF is observed [64, 65]. Subsequent analyses further revealed disparities in kidney donation patterns between the two risk groups. Specifically, the high‐risk group comprised a greater number of DCD donors (60), whereas the low‐risk group had more living and DBD donors (66 and 31, respectively; *p*  < 0.0001; Figure 6J). Furthermore, an examination of subtype distribution in the two risk groups indicated that cluster A was significantly associated with the high‐risk group compared to the low‐risk group (75 vs. 12, respectively; *p*  < 0.0001), thereby affirming our earlier hypotheses (Figure 6J). A Sankey diagram was employed to visually represent the composition of donor types, graft function, subtypes, and DGF risk groups in IRI samples (Figure 6K). This procedure revealed a higher infiltration of myeloid immune cells in the high‐risk group, a finding corroborated by multiple algorithms. Additionally, the high‐risk group had a greater proportion of kidneys from DCD donors and included more cluster A members, further reinforcing the association between donor type and DGF occurrence. These results highlight the critical role of myeloid immune cells and donor type in driving immune‐mediated damage and graft dysfunction in patients at high risk for DGF.

### 3.7. Construction of DGF‐Related TFs‐Hub DE‐IRGs and TFs‐miRNAs Hub DE‐IRGs Regulatory Networks

**Figure 7:**
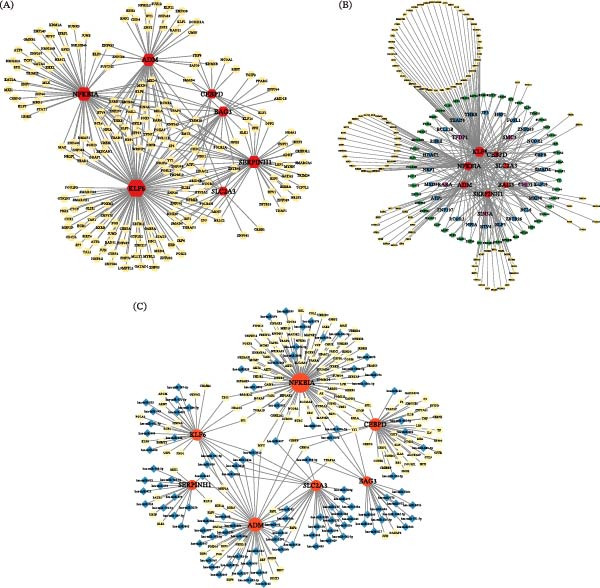
Construction of DGF‐related TF‐hub DE‐IRGs and TF‐miRNA hub DE‐IRGs regulatory networks. (A) and (B) present the same TF‐hub DE‐IRG network outcome with varying visual inclinations for optimal presentation. The interaction counts for KLF6, NFKBIA, ADM, SERPINH1, BAG3, CEBPD, and SLC2A3 are 104, 65, 48, 46, 24, 15, and 11, respectively, with RARA, TFDP1, SIN3A, SMC3, and CHD1 regulating up to four hub DE‐IRGs. (C) TFs‐miRNAs hub DE‐IRGs regulatory network demonstrating the interaction counts between hub DE‐IRGs and miRNAs are NFKBIA (102), ADM (55), CEBPD (42), KLF6 (31), SLC2A3 (30), SERPINH1 (19), and BAG3 (19), with hsa‐miR‐181a, hsa‐miR‐181b, hsa‐miR‐181c, hsa‐miR‐181d, and MEF2A, YY1 interacting with up to three hub DE‐IRGs. Purple hexagons represent TFs interacting with four hub DE‐IRGs; blue hexagons represent TFs interacting with three DE‐IRGs; green hexagons represent TFs interacting with two hub DE‐IRGs; yellow hexagons represent TFs interacting with one hub DE‐IRG; yellow ellipses represent TFs interacting with one hub DE‐IRG; and blue diamonds represent miRNAs interacting with hub DE‐IRGs.

To uncover significant transcriptional alterations and gain deeper insights into the hub DE‐IRGs, we adopted a network‐based strategy to decipher the regulatory roles of TFs and miRNAs. TFs are proteins that bind specifically to DNA sequences, thereby orchestrating the transcriptional process from DNA to messenger RNA [66]. In this study, a total of 196 TFs were identified to interact with the seven DGF‐related hub DE‐IRGs using NetworkAnalyst 3.0, as illustrated in Figure 7A,B. The network interaction results are summarized in Supporting Information 1: Table S5. Both Figure 7A,B present the same network outcome with varying visual inclinations for optimal presentation. The size of the hub DE‐IRGs (depicted as red hexagons) corresponds to the number of connections each gene has with other TFs in the network. Based on the interaction count within this network, we ranked the hub DE‐IRGs as follows: KLF6 (104), NFKBIA (65), ADM (48), SERPINH1 (46), BAG3 (24), CEBPD (15), and SLC2A3 (11). Notably, TFs such as RARA, TFDP1, SIN3A, SMC3, and CHD1 (depicted as purple hexagons) exhibited greater significance, as they interacted with up to four hub DE‐IRGs in this regulatory network, while TFs denoted by blue hexagons interacted with up to three (24), green hexagons with two (50), and yellow hexagons with one (117; Figure 7B).

miRNAs are known to act through the miRNA‐induced silencing complex to bind target mRNAs, triggering either translational repression or deadenylation [67, 68]. In our study, we sought to predict miRNAs capable of interacting with hub DE‐IRGs using NetworkAnalyst 3.0, thereby constructing a coregulatory network involving TFs, miRNAs, and hub DE‐IRGs. This network encompassed a total of 107 miRNAs and 151 TFs (Figure 7C). The network interaction results are summarized in Supporting Information 1: Table S6. The network analysis revealed a hierarchy of hub DE‐IRGs based on their interaction number with miRNAs, with the following ranking: NFKBIA (102), ADM (55), CEBPD (42), KLF6 (31), SLC2A3 (30), SERPINH1 (19), and BAG3 (19). Furthermore, we identified significant hub miRNAs and TFs within this coregulatory network, including hsa‐miR‐181a, hsa‐miR‐181b, hsa‐miR‐181c, and hsa‐miR‐181d (miRNAs), and MEF2A and YY1 (TFs), all of which interacted with up to three hub DE‐IRGs. This procedure constructed regulatory networks involving TFs and miRNAs that interact with seven hub DE‐IRGs, revealing significant transcriptional alterations linked to DGF, with the identification of key TFs and miRNAs. These networks highlight the complex regulatory mechanisms underlying immune response and graft dysfunction, offering insights into potential therapeutic targets.

### 3.8. Compounds Prediction for DGF‐Related Hub DE‐IRGs and Molecular Docking Simulations

**Figure 8:**
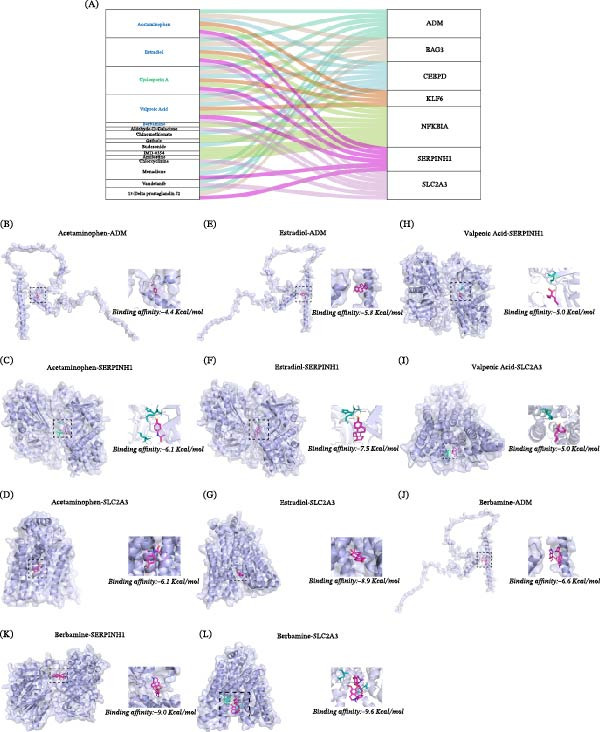
Screening predicted compounds for DGF‐related hub DE‐IRGs and molecular docking simulations. (A) Compounds Sankey diagram illustrating the top 15 predicted compounds among 516 potential ones. These were selected based on a criterion of adjusted *p*‐value < 0.05 and ranked by odds ratio. Blue labeled predicted compounds present the top three ranked potential compounds, excluding cyclosporin A. (B–D) Molecular docking plot showing the docking results between acetaminophen and ADM (B), SERPINH1 (C), and SLC2A3 (D), with the binding energies of −4.4, −6.1, and −6.1, respectively. (E–G) Molecular docking plot showing the docking results between estradiol and ADM (E), SERPINH1 (F), and SLC2A3 (G), with the binding energies of −5.8, −7.5, and −8.9, respectively. (H–I) Molecular docking plot showing the docking results between valproic acid and SERPINH1 (H), and SLC2A3 (I), with the binding energies of −5.0 for both. (J–L) Molecular docking plot showing the docking results between berbamine and ADM (J), SERPINH1 (K), and SLC2A3 (L), with the binding energies of −6.6, −9.0, and −9.6, respectively. Yellow dotted lines indicate the polar contacts between predicted compounds and specific amino acids, such as HIS‐209, ALA‐261, and SER‐121 in (C), HIS‐209, and ALA‐261 in (F), SER‐265 in (H), THR‐293 in (I); and GLU‐175 and TYR‐290 in (L).

Having explored the potential role of hub DE‐IRGs in predicting the occurrence of DGF and constructing the regulatory networks surrounding these genes, it became evident that these hub genes could serve as potential targets for drug intervention. Leveraging Enrichr with transcriptome signatures from the DSigDB database, we predicted a total of 516 compounds, employing a criterion of adjusted *p*‐value < 0.05 and ranking them by Odds Ratio (Supporting Information 1: Table S7). Following a meticulous search and identification for compounds with potential immunosuppressive capacity, we screened the top 15 predicted compounds, considering them as promising candidates for DGF treatment in subsequent analyses. These 15 potential therapeutic agents encompassed acetaminophen, estradiol, cyclosporin A, valproic acid, berbamine, aldehydo‐D‐galactose, chinomethionate, osthole, budesonide, IMD‐0354, amifostine, chlorcyclizine, menadione, vandetanib, and 15‐delta prostaglandin J2. They were illustrated alongside their respective targeting hub DE‐IRGs (Figure 8A).

Furthermore, three genes were selected for subsequent molecular docking analysis due to their relative adverse coefficients within our preconstructed DGF predictive model (ADM 0.650, SERPINH1 0.366, and SLC2A3 0.137). This selection aimed to predict the binding mode of these three hub DE‐IRGs with the top four ranked potential compounds, namely, acetaminophen, estradiol, valproic acid, and berbamine. Notably, although berbamine targets only NFKBIA in the DSigDB database analysis, we included it in subsequent docking simulations with the other three hub DE‐IRGs to assess their docking potential. This decision was based on the high combined score with NFKBIA and the close interaction network among the DE‐IRGs (Supporting Information 1: Table S7; Figure 3A). Cyclosporin A was excluded from this phase of the analysis due to its well‐established general antirejection effects. The prevailing notion suggests that a lower binding energy between ligands (predicted compounds) and receptors (hub DE‐IRGs) signifies stronger binding stabilization.

A total of 11 predicted compounds and hub DE‐IRGs’ molecular docking results were illustrated and depicted in both macro and micro views, except for one result from valproic acid, which exhibited a negative docking result with ADM (−3.5 kcal/mol; Figure 8B–L). The binding energy results for acetaminophen with ADM, SERPINH1, and SLC2A3 were −4.4, −6.1, and −6.1 kcal/mol, respectively (Figure 8B–D). Notably, acetaminophen formed four polar contacts with three amino acids (HIS‐209, ALA‐261, and SER‐121) when binding SERPINH1 (Figure 8C). Estradiol demonstrated even more promising results, achieving binding energies of −5.8, −7.5, and −8.9 kcal/mol with ADM, SERPINH1, and SLC2A3, respectively, with the latter two meeting the criteria for strong binding affinity, suggesting estradiol’s superior potential for DGF treatment (Figure 8E–G). Interestingly, estradiol formed three polar contacts with the same two amino acids (HIS‐209 and ALA‐261) in SERPINH1 as acetaminophen, hinting at potential similarities in their active binding structures (Figure 8F). Valproic acid, despite exclusion from further analysis due to its weak binding with ADM, displayed binding energies of −5.0 kcal/mol with SERPINH1 and SLC2A3 for both, with the formation of polar contacts at SER‐265 in SERPINH1 and THR‐293 in SLC2A3 (Figure 8H,I). Berbamine achieved binding energies of −6.6, −9.0, and −9.6 kcal/mol with ADM, SERPINH1, and SLC2A3, respectively, with the latter two meeting the criteria for strong binding affinity (Figure 8J–L). Meanwhile, berbamine formed two polar contacts with the two amino acids (GLU‐175 and TYR‐290) in SLC2A3 (Figure 8L). Therefore, based on the molecular docking simulations and identifications, these four predicted compounds may be the most promising compounds for treating DGF in KTx patients, with berbamine emerging as the top‐ranked candidate. Further investigations into the pharmacological effects of these predicted compounds are warranted. This procedure identified 15 predicted compounds targeting DGF‐related hub DE‐IRGs, with acetaminophen, estradiol, valproic acid, and berbamine selected for molecular docking simulations. Docking results revealed strong binding affinities of acetaminophen, estradiol, and berbamine with key hub DE‐IRGs (SERPINH1, SLC2A3, and ADM), suggesting their potential for DGF treatment.

### 3.9. Molecular Docking Analysis Reveals Interactions Between Common Immunosuppressants and Key DGF‐Related Hub DE‐IRGs in Renal Transplantation

**Figure 9:**
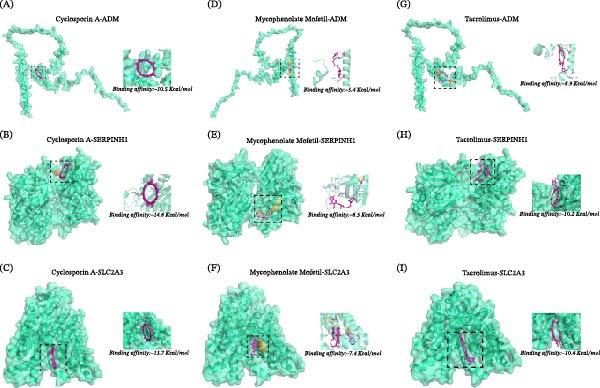
Molecular docking simulations for common immunosuppressants in postrenal transplantation. (A–C) Plots showing the docking results between cyclosporin A and ADM (A), SERPINH1 (B), and SLC2A3 (C), with the binding energies of −10.5, −14.6, and −13.7, respectively. (D–F) Plots showing the docking results between MMF and ADM (D), SERPINH1 (E), and SLC2A3 (F), with the binding energies of −5.4, −6.3, and −7.4, respectively. (G–I) Plots showing the docking results between tacrolimus and ADM (G), SERPINH1 (H), and SLC2A3 (I), with the binding energies of −8.9, −10.2, and −10.4, respectively. Yellow dotted lines indicate the polar contacts between immunosuppressants and specific amino acids, such as GLN‐368 in (B), TRP‐34 and ARG‐23 in (D), LYS‐213, ARG‐239, and ARG‐222 in (E), GLN‐170 and TRP‐305 in (F); and GLN‐123, ASP‐133, and VAL‐135 in (G). MMF, mycophenolate mofetil.

Following the identification of four potential therapeutic agents for DGF in KTx patients, we sought to explore the molecular interactions between commonly used immunosuppressants and key DGF‐related hub DE‐IRGs (SERPINH1, SLC2A3, and ADM). Cyclosporin A, as highlighted by the Enrichr analysis, alongside with the other two common immunosuppressants, MMF and tacrolimus, was subjected to molecular docking. Cyclosporin A exhibited binding energies of −10.5, −14.6, and −13.7 kcal/mol with ADM, SERPINH1, and SLC2A3, respectively, demonstrating a strong binding affinity (Figure 9A–C). Notably, its interaction with SERPINH1 involved a polar contact with GLN‐368 (Figure 9B). MMF showed binding energies of −5.4, −6.3, and −7.4 kcal/mol with ADM, SERPINH1, and SLC2A3, forming polar contacts with amino acids TRP‐34 and ARG‐23 in ADM; LYS‐213, ARG‐239, and ARG‐222 in SERPINH1; and GLN‐170 and TRP‐305 in SLC2A3 (Figure 9D–F). Tacrolimus yielded binding energies of −8.9, −10.2, and −10.4 kcal/mol with ADM, SERPINH1, and SLC2A3, respectively, forming polar contacts with GLN‐123, ASP‐133, and VAL‐135 in ADM (Figure 9G–I).

Collectively, these results indicate that cyclosporin A exhibits the strongest binding affinity across all three immunosuppressants, followed by tacrolimus and MMF. These findings provide novel insights into the therapeutic target profiles of commonly used immunosuppressants in KTx patients, laying a foundation for future pharmacological studies. By revealing the molecular interactions between key immunosuppressants (cyclosporin A, MMF, and tacrolimus) and critical DGF‐related DE‐IRGs (SERPINH1, SLC2A3, and ADM), this procedure enhances our understanding of their potential mechanisms of action in the management of DGF.

### 3.10. Discovery and Investigation of Survival‐Related DE‐IRGs for Long‐Term Graft Survival Prediction

**Figure 10:**
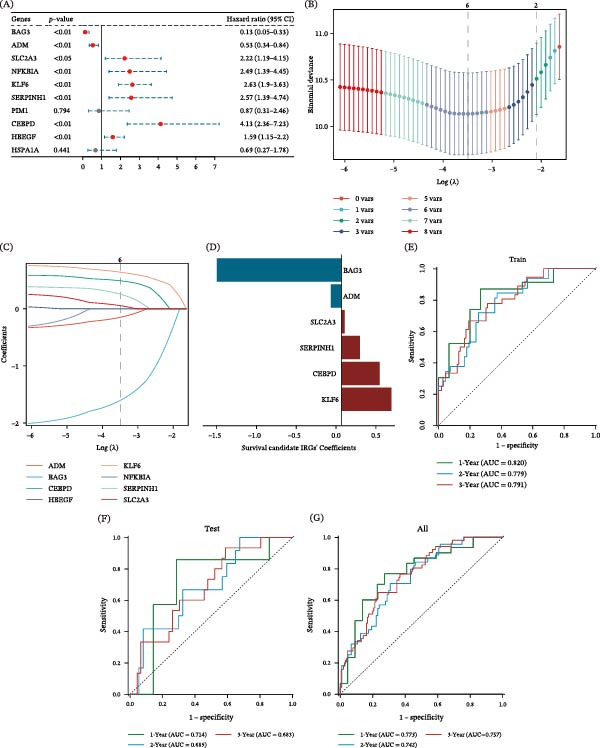
Establishment of graft survival model. (A) Forest plot displaying the results of univariate Cox regression analysis of the 10 RF/SVM‐RFE feature DE‐IRGs, with eight of them achieving statistical significance. (B, C) Six survival‐related hub DE‐IRGs identified via a tenfold cross‐validated LASSO regression algorithm, indicated by the minimum binomial deviance at lambda.min. (D) Coefficients of the survival‐related hub DE‐IRGs. (E–G) Time‐dependent ROC plot evaluating the performance of the long‐term graft survival predictive model, with 1–, 2‐, 3‐ year AUC values of 0.820, 0.779, and 0.791 in the training set (E), 1–, 2‐, and 3‐ year AUC values of 0.714, 0.685, and 0.683 in the testing set (F), and 1–, 2‐, and 3‐ year AUC values of 0.773, 0.742, and 0.757 in all sets (G). AUC, area under the curve.

Considering the significant implications of DGF in KTx patients and its intricate connection with graft outcomes, we embarked on a detailed construction of a robust predictive model for graft survival. Utilizing univariable Cox regression analysis, we screened long‐term survival‐related DE‐IRGs (CEBPD, KLF6, NFKBIA, BAG3, SLC2A3, SERPINH1, HBEGF, and ADM) based on the 10 RF/SVM‐RFE feature DE‐IRG samples derived from the GSE21374 dataset. The results of this analysis are depicted in the forest plot presented in Figure 10A.

**Figure 11:**
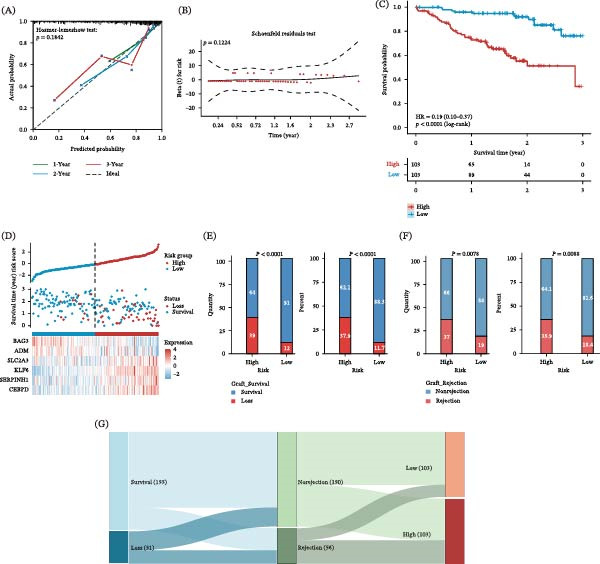
Clinical correlations of the two survival prognosis risk groups. (A) Calibration curve plot demonstrating relative alignment between the 1‐year and 2‐year curves with the ideal curve, with slight instability in the 3‐year curve. (B) Schoenfeld Residuals Test plot validated the PH assumption. (C) K–M survival plot showing significant disparity in graft survival prognosis between the two risk groups. (D–E) High‐risk recipients were significantly more prone to experience allograft loss compared to low‐risk recipients. (F) Stacked bar charts showing more incidence of graft rejection in the high‐risk group. (G) Sankey diagram summarizing the patient stratification process based on clinical outcomes, such as graft survival or rejection, and subsequent risk scores. HR, hazard ratio; PH, proportional hazard.

We allocated all recipients in the GSE21374 dataset into two sets (in a 7:3 ratio) for training and testing purposes. Leveraging the eight prognostic DE‐IRGs, we applied the LASSO regression algorithm to the training set to identify pivotal survival‐related DE‐IRGs. The optimal number of hub genes was determined to be six, guided by the minimum binomial deviance (Figure 10B). Consequently, six survival‐related hub DE‐IRGs (BAG3, ADM, SLC2A3, SERPINH1, CEBPD, and KLF6) were selected for constructing the graft survival predictive model, along with their corresponding regression coefficients (−1.563, −0.133, 0.042, 0.232, 0.481, and 0.629, respectively) (Figure 10B–D; Supporting Information 1: Table S8). Following model construction, we computed risk scores for each IRI sample using the formula: Riskscore = −1.563 × BAG3 expression −0.133 × ADM expression + 0.042 × SLC2A3 expression + 0.232 × SERPINH1 expression + 0.481 × CEBPD expression + 0.629 × KLF6 expression. Subsequently, we stratified all samples into high‐risk and low‐risk groups based on the median risk score. Evaluation of the model’s performance through time‐dependent ROC curves revealed satisfactory AUC values for 1–3 years of survival across the training set (0.820, 0.779, and 0.791), internal testing set (0.714, 0.685, and 0.683), and all sets (0.773, 0.742, and 0.757), respectively (Figure 10E–G). Additionally, calibration curve analysis demonstrated relative alignment between the 1‐year and 2‐year curves with the ideal curve, albeit with slight instability in the 3‐year curve, indicating good agreement between predicted and actual probabilities. This finding was further supported by the Hosmer–Lemeshow test, which yielded a *p*‐value of 0.1842, suggesting no statistical difference between predicted and actual probabilities (Figure 11A). Upon grouping patients based on median risk scores, we observed significant differences between the high‐risk (*n* = 103) and low‐risk groups (*n* = 103). Kaplan–Meier analysis illustrated notably worse survival outcomes in the high‐risk group as the year progressed (*p*  < 0.0001; Figure 11C). Cox PH regression analysis revealed an 80% decrease in survival hazards when comparing the low‐risk group to the high‐risk group (HR = 0.19 [0.10–0.37], *p*  < 0.001), with the PH assumption validated by the Schoenfeld Residuals Test (*p* = 0.1224; Figure 11B, C). Notably, high‐risk recipients were significantly more prone to experience allograft loss compared to low‐risk recipients (39 vs. 12, 37.9% vs. 11.7%, *p*  < 0.0001; Figure 11E), mirroring the occurrence of graft rejection (37 vs. 19, *p*  = 0.0078, 35.9% vs. 18.4%, *p* = 0.0088; Figure 11F). Finally, a Sankey diagram summarizes the patient stratification process based on risk scores and subsequent clinical outcomes (Figure 11G). This procedure developed a predictive model for long‐term graft survival in kidney transplant patients by identifying six hub DE‐IRGs linked to survival outcomes. Using risk scores based on these genes, patients were stratified into high‐ and low‐risk groups, with the high‐risk group showing significantly worse survival and an increased risk of graft loss and rejection. The model demonstrated good predictive performance and offers valuable insights for identifying patients at higher risk of poor graft survival.

### 3.11. Validation and Investigation of Hub DE‐IRGs Clinical Significance for Renal Allograft Function

**Figure 12:**
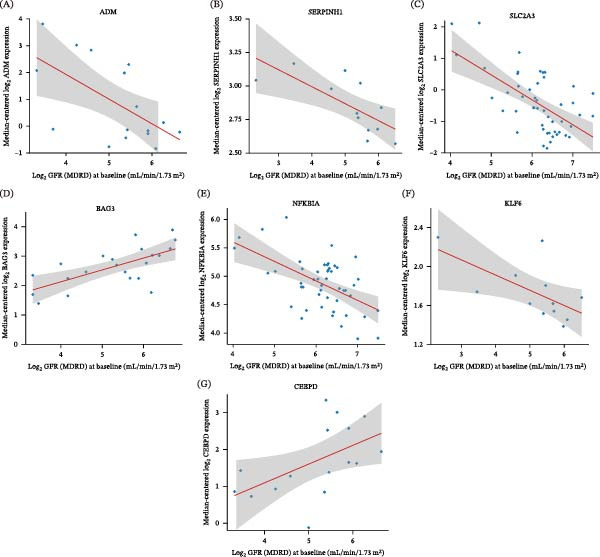
Validation of clinical significance of hub DE‐IRGs correlated with renal allograft function. (A–C, E–F) Scatter plots illustrating significant inverse correlations between the mRNA expression levels of ADM (A), SERPINH1 (B), SLC2A3 (C), NFKBIA (E), and KLF6 (F) and the estimated GFR. (D, G) Scatter plots demonstrating positive associations of BAG3 (D) and CEBPD (G) expression with renal function parameters. All data were retrieved from the Nephroseq v5 database. GFR, glomerular filtration rate; MDRD, Modification of Diet in Renal Disease.

To validate the clinical significance of the seven identified hub DE‐IRGs, we analyzed their correlation with the renal allograft function using the Nephroseq v5 database. As illustrated in Figure 12, the expression levels of ADM (Figure 12A), SERPINH1 (Figure 12B), SLC2A3 (Figure 12C), NFKBIA (Figure 12E), and KLF6 (Figure 12F) exhibited significant negative correlations with the estimated GFR (MDRD). This inverse relationship indicates that elevated intragraft expression of these targets is closely linked to a compromised filtration capacity. In contrast, BAG3 (Figure 12D) and CEBPD (Figure 12G) displayed converse associations, showing positive correlations with functional parameters. Collectively, these findings underscore the pathological relevance of the identified hub genes, linking their dysregulated expression to the deterioration of long‐term allograft function.

### 3.12. In Vivo Validation of Hub DE‐IRGs in the Mouse UIRI Model

**Figure 13:**
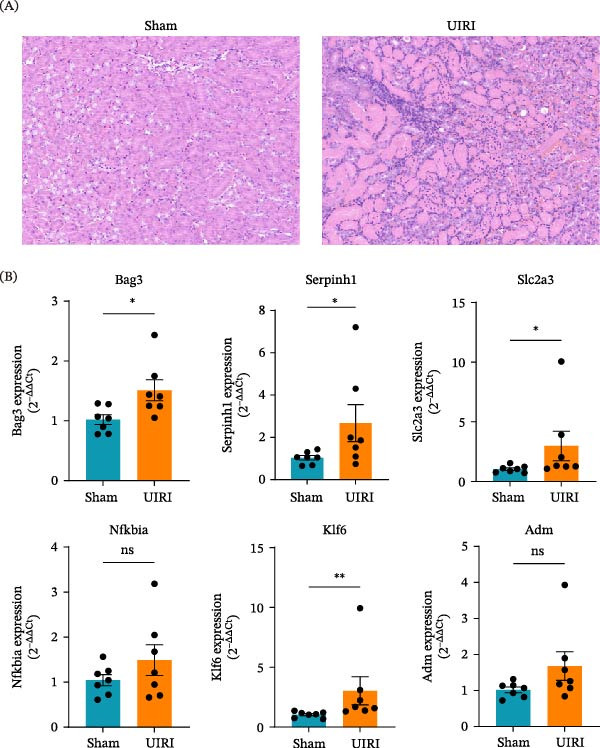
Validation of hub DE‐IRGs and renal pathological changes in the mouse model. (A) Representative H&E staining images of renal tissues from the Sham and UIRI groups, demonstrating severe tubular damage and inflammatory cell infiltration in the injured kidneys. (B) qRT‐PCR analysis of the mRNA expression levels of the six hub genes (*Nfkbia*, *Bag3*, *Serpinh1*, *Klf6*, *Slc2a3*, and *Adm*) following UIRI compared to the Sham group. BAG3, SERPINH1, KLF6, and SLC2A3 were significantly upregulated in the UIRI group, while NFKBIA and ADM showed no significant differences. All data points in bar charts represent mean and SEM (*n* = 7 biological replicates per group). Statistical significance was determined using the Mann–Whitney *U* test. ns, not significant;  ^∗^ *p*  < 0.05,  ^∗∗^ *p*  < 0.01. DE‐IRGs, differentially expressed immune infiltration‐related genes; H&E, hematoxylin and eosin; qRT‐PCR, quantitative real‐time PCR; SD, standard deviation; UIRI, unilateral ischemia‐reperfusion injury.

To further validate six of the seven identified hub DE‐IRGs, we analyzed their gene expression in mouse models. We first evaluated the pathological changes in the kidney tissues using H&E staining (Figure 13A). Compared with the Sham group, H&E staining of the renal tissues in the UIRI group showed severe pathological changes, including obvious tubular damage, loss of normal tissue architecture, and massive inflammatory cell infiltration.

Following the UIRI models, the mRNA expression of the six hub genes was validated in mouse renal tissues by qRT‐PCR (Figure 13B). The evaluated genes included Nfkbia, Bag3, Serpinh1, Slc2a3, Klf6, and Adm. The results showed that the expression levels of Bag3, Serpinh1, Klf6, and Slc2a3 after UIRI were significantly upregulated compared with the Sham group. Specifically, Klf6 showed highly significant upregulation ( ^∗∗^ *p*  < 0.01), whereas Bag3, Serpinh1, and Slc2a3 were significantly increased ( ^∗^ *p*  < 0.05). In contrast, the mRNA expression levels of Nfkbia and Adm showed no significant difference between the UIRI and Sham groups. The lack of significant upregulation for Nfkbia and Adm at 24 h postreperfusion may reflect species differences or transient expression peaks (see Section 4). Overall, these results successfully validated the significant upregulation of Bag3, Serpinh1, Klf6, and Slc2a3 in renal tissues following UIRI.

## 4. Discussion

KTx stands as the ultimate treatment option for patients with ESKD. However, the procedure often leads to a complication known as IRI caused by the interruption and subsequent reintroduction of blood supply to organs. The critical role of immune cell infiltration and subsequent inflammatory responses in renal IRI pathology is well‐documented [23–27]. A multitude of studies underscore that mitigating this process can lead to improved kidney function. The process of leukocyte recruitment from the bloodstream into injured tissues is multistep and varies across different organs. In postischemic kidneys, an immune response is triggered by the resident immune cells and is further amplified by the rapid influx of immune cells through the compromised endothelium. TLRs, adhesion molecules, and DAMPs released from dying cells play essential roles in facilitating the recruitment and activation of diverse immune cell types, particularly during the early phase of injury. The activation of the complement system and the heightened production of proinflammatory cytokines and chemokines serve as crucial promoters of leukocyte infiltration into the postischemic kidney. Major effector cells of the innate immune system, such as macrophages, DCs, and NK cells, are intricately involved in the pathogenesis of renal injury following IRI. Meanwhile, T cells, the principal effector cells of the adaptive immune system, substantially contribute to renal injury development from the early to late phases of injury. Furthermore, plasma cells appear to participate in the tubular damage process during the late phase of injury [21, 22]. DGF stands as a common early complication associated with renal IRI during renal transplantation [21, 69, 70]. It often leads to renal dysfunction, graft loss, and, in severe cases, mortality, underscoring the significant clinical implications of immune‐mediated renal injury in this context [7, 12–14]. While the roles of immune cells have been reported over the past decades, there is currently a lack of comprehensive analysis regarding potential biomarkers associated with immune infiltration in IRI following KTx.

In our study, employing various ML methodologies on transcriptomic data from KTx patients experiencing renal IRI sourced from GEO databases, we identified seven hub DE‐IRGs significantly associated with the intricate interplay between immune infiltration processes and DGF. These pivotal genes, namely, ADM, SERPINH1, SLC2A3, BAG3, NFKBIA, KLF6, and CEBPD, exhibited notable correlations with the majority of DI‐ICs. Among these hub genes, BAG3, ADM, SLC2A3, SERPINH1, CEBPD, and KLF6 have also been identified as survival‐related hub DE‐IRGs. Extensive prior research has underscored the intricate involvement of all seven hub genes in immune infiltration or inflammatory biological processes. ADM, a multifunctional peptide, is expressed by various cell types, including endothelial cells, macrophages, T cells, mast cells, and DCs. Its primary biological effect involves vasodilation and maintenance of vascular barrier function [71]. While ADM’s anti‐inflammatory properties have been widely acknowledged [71–74], recent studies have unveiled its proinflammatory roles [75, 76]. For instance, Han et al. [75] elucidated ADM’s proinflammatory effect by directly promoting the expansion and activation of group 2 innate lymphoid cells, leading to IL‐5 secretion and subsequent lung airway inflammation accompanied by enhanced eosinophilia in lung tissues. Similarly, Kong et al. [76] demonstrated how ADM induces the differentiation of IFN‐γ‐producing T cells through the phosphorylation of AKT and STAT3 while also stimulating macrophages to produce IL‐12, thus promoting IFN‐γ‐producing T‐cell responses, which collectively contributes to gastritis during *H. pylori* infection. SERPINH1, localized in the endoplasmic reticulum, functions as a collagen‐specific molecular chaperone, pivotal in collagen biosynthesis. Beyond its role in collagen synthesis, SERPINH1 exhibits regulatory effects on cell migration, as evidenced in highly invasive breast cancer cells, where it interacts with nonmuscle myosin IIA [77]. Furthermore, Wang et al. [60] uncovered SERPINH1’s significant regulatory impact on immune cell infiltration, which aligns with our observations. Likewise, Abe et al. [78] identified SERPINH1’s capability to recruit macrophages in renal allograft tissues. SLC2A3, also known as GLUT3, serves as a crucial mediator of glucose import across the plasma membrane and transport across the blood‐brain barrier [79]. Recent studies have shed light on its role in promoting macrophage infiltration through glycolysis reprogramming in gastric cancer [61]. Hochrein et al. [80] elucidated how SLC2A3‐dependent glucose uptake regulates a metabolic‐transcriptional circuit controlling the expression of inflammatory cytokines in Th17 cells. Similarly, Huang et al. [81] identified SLC2A3 as a key downstream effector of LPCAT1, enhancing keratinocyte glycolysis and thereby fostering proproliferative and proinflammatory effects. BAG3 serves as an antiapoptotic protein while also influencing cell adhesion and motility regulation [82–84]. Avinery et al. [85] recently highlighted its role as a HSP70‐bound cochaperone, which, in conjunction with the TF LITAF, regulates tumor macrophage infiltration. This regulation occurs through targeting inflammatory cytokines and chemokines expression in macrophages [85]. Additionally, Gong et al. [59] identified BAG3 as a novel prognostic biomarker in kidney renal clear cell carcinoma due to its significant regulatory impact on immune cell infiltration. NFKBIA encodes a protein that interacts with REL dimers to inhibit NF‐kappa‐B/REL complexes, which play crucial roles in immune infiltration and inflammatory responses [62, 86, 87]. In our study, the observed upregulation of NFKBIA in the post‐IRI group suggests potential protective properties against inflammation. KLF6, a member of the Kruppel‐like factor family, is involved in various renal cellular processes, including renal fibrosis and interstitial inflammation [88]. Recent studies have highlighted its role in comodulating with RUNX1 to control neutrophil maturation, a process critical for neutrophil recruitment ability [89]. Additionally, KLF6 regulates macrophage inflammatory gene expression by modulating the functions of NF‐κB and PPARγ [90]. The final hub DE‐IRG, CEBPD, encodes the C/EBPδ TF, which serves as a key regulator of inflammatory responses [91]. Upon TLR4‐induced macrophage activation, NF‐κB binds to the CEBPD promoter, initiating CEBPD transcription. Subsequently, CEBPD binds to the IL‐6 promoter and cooperates with NF‐κB to fully activate IL‐6 transcription. Additionally, CEBPD acts as an amplifier of NF‐κB‐mediated transcription, distinguishing between transient and persistent TLR4 signals and facilitating the sustained expression of the inflammatory response [92]. Utilizing the seven DGF‐related hub DE‐IRGs, consensus clustering enabled the identification of two distinct IRI subtypes. Patients in cluster A appeared to undergo a more severe immune cell infiltration process and subsequent injury, evidenced by their stronger association with DCD donors and DGF occurrence [93]. Comparing cluster A to cluster B, upregulation of BAG3, SLC2A3, and SERPINH1 was observed in cluster A, whereas KLF6, NFKBIA, and CEBPD were downregulated. This suggests that the former three genes may have a more detrimental impact on immune cell‐induced renal allograft injury.

Multiple studies have revealed intricate interactions between immune and renal cells contributing to transplant renal IRI and subsequent allograft rejection [22, 94–98]. Our research corroborates these findings, demonstrating elevated levels of up to 16 DI‐ICs, including activated CD4 T cells, activated CD8 T cells, activated DCs, effector memory CD4 T cells, effector memory CD8 T cells, eosinophils, gamma delta T cells, mast cells, memory B cells, monocytes, natural killer T cells, neutrophils, regulatory T cells, T follicular helper cells, type 1 T helper cells, and type 17 T helper cells. Utilizing ML methods, we identified three core DI‐ICs that are most closely associated with DGF occurrence: activated CD8 T cells, activated DCs, and effector memory CD4 T cells. Previous studies align with our findings, highlighting the comprehensive involvement of DCs in the IRI process and their promotion of harmful immune activations [94, 97]. Activated DCs recruit immature or memory T cells attracted by the CCR7 gradient to present the allografts, inducing acute rejection [98]. Activated CD8 T cells also play a crucial role in allograft rejection following KTx [96]. Furthermore, the expression levels of the seven DGF‐related hub biomarkers positively correlate with the extent of infiltration by core immune cells in the kidney samples affected by DGF. These results suggest that these hub biomarkers are likely involved in regulating the immune response that promotes DGF occurrence. Drawing upon the DGF‐related hub DE‐IRGs and survival‐related DE‐IRGs along with their respective coefficients, we constructed predictive models for DGF and graft loss, respectively. Remarkably, the DGF predictive models exhibited a nearly ideal calibration plot and outperformed existing DGF prediction tools with AUC = 0.832 and 0.975, such as the Irish score [15]. Patient stratification based on risk scores revealed that the DGF‐related high‐risk group exhibited a higher abundance of myeloid immune cell infiltration, particularly macrophages and neutrophils. Notably, heterogeneity in immune cell quantification was observed across algorithms. As shown in Figure 6, discrepancies existed, particularly regarding neutrophils (e.g., between ssGSEA and xCELL). These variations likely stem from distinct methodological principles (rank‐based enrichment vs. deconvolution), a phenomenon documented in benchmark studies [99, 100]. Consequently, these findings represent algorithmic estimations. While offering valuable directional insights into the DGF immune microenvironment, they require future confirmation such as flow cytometry or multiplex immunohistochemistry on independent clinical tissue samples. This high‐risk group also showed a poorer prognosis, characterized by a higher incidence of DGF. Notably, there was a significant overlap between the DGF‐related high‐risk group and cluster A. Likewise, the survival‐related high‐risk group demonstrated unfavorable outcomes, including a higher incidence of allograft rejection and a shorter survival time. These findings underscore the significance of hub DE‐IRGs in the early prediction of graft dysfunction and subsequent graft outcomes, offering novel insights into future biological diagnosis and therapeutic targeting.

TFs are pivotal proteins that intricately regulate the transcriptional process from DNA to messenger RNA [66, 101], thereby governing various biological pathways, including immune responses [102]. In our investigation, we constructed the regulatory network of DGF‐related TFs and hub DE‐IRGs, identifying five TFs—RARA, TFDP1, SIN3A, SMC3, and CHD1—as potential keystones in this regulatory cascade. Notably, recent studies have shed light on the crucial roles of these TFs in immune regulation pathways, corroborating our findings. For instance, RARA deletion has been associated with increased renal proximal tubule secretion of transforming growth factor β1, inflammation, interstitial fibrosis, CKD, and reduced kidney function [103]. Likewise, the inhibitory effects of TFDP1 on inflammation and apoptosis, coupled with its proliferative promotion, have been elucidated by Li et al. [104]. Furthermore, SIN3A has been implicated in rheumatoid arthritis, with 15 other specific CG sites identified as biomarkers for this condition [105]. Additionally, SMC3 has been shown to play a pivotal role in B‐cell to plasma cell transitions and to restrain the malignant transformation of germinal center B cells [106]. Finally, CHD1 deletion has been linked to significant remodeling of the tumor microenvironment, characterized by reduced myeloid‐derived suppressor cells and increased CD8+ T cells [107]. miRNAs exert their regulatory functions through the miRNA‐induced silencing complex, which binds to target mRNAs, resulting in translational repression or deadenylation [67, 68]. In our exploration of miRNA‐mediated regulation of DGF‐related hub DE‐IRGs, we constructed a coregulatory network involving TFs, miRNAs, and hub DE‐IRGs. Among the identified core miRNAs were hsa‐miR‐181a, hsa‐miR‐181b, hsa‐miR‐181c, and hsa‐miR‐181d, along with two core TFs, MEF2A and YY1. The vertebrate miR‐181 family encompasses six mature miRNAs: miR‐181a‐1, miR‐181a‐2, miR‐181b‐1, miR‐181b‐2, miR‐181c, and miR‐181d. Previous research has underscored the crucial role of the miR‐181 family in regulating normal hematopoietic lineage differentiation, particularly in the development of B, T, and NK cells [108]. Studies by Cilenti et al. [109] have identified MEF2A as a pivotal regulator of the inflammatory epigenome in macrophages, particularly at PGE2‐sensitive enhancers. Deletion of MEF2A results in the functional inactivation of these regions, impairing the inflammatory induction of IFN‐b upon exposure to innate immune stimuli or pathogens [109]. Recently, YY1 has been implicated in the anti‐inflammatory effects of quercetin on diabetic nephropathy‐associated tubulointerstitial fibrosis. It was found that YY1 plays a crucial role in mitigating tubulointerstitial inflammation mediated by the IL‐6/STAT‐3 pathway [110], contrasting its previously identified profibrotic role [111]. Collectively, these findings underscore the association of these TFs and miRNAs with specific immune regulation pathways, thereby aligning with our discoveries.

To explore potential strategies for mitigating the occurrence of DGF and its subsequent detrimental effects on renal function, we employed the Enrichr system and molecular docking identification to predict potential compounds and explore the interactions between common immunosuppressants and key hub DE‐IRGs. Our analysis yielded a total of 516 candidate compounds, from which we prioritized the top 15 predicted compounds and identified four core compounds targeting the most negatively associated genes: acetaminophen, estradiol, valproic acid, and berbamine. Recent research has shed light on acetaminophen’s potential as a suppressor of antitumor immunity, notably by inducing the expansion of Tregs and IL‐10 production, a key mediator of Treg‐induced immune suppression [112]. Given that autologous Tregs have demonstrated efficacy in minimizing immunosuppression doses in post‐KTx [113], acetaminophen aligns with our study by offering its Tregs‐enhancing potential. Similarly, estradiol has been extensively investigated for its ability to enhance the suppressive function of Tregs [114]. Early topical estradiol therapy has shown promising results in significantly reducing the occurrence of chronic graft‐versus‐host disease of the anogenital zone in patients undergoing hematopoietic cell transplantation [115]. Crucially, 17β‐estradiol attenuates renal IRI by inhibiting the TGF‐βRI/SMAD pathway via estrogen receptor α, thereby reducing tissue apoptosis and fibrosis [116]. Valproic acid, widely used as an antiepileptic and mood stabilizer, has recently demonstrated anti‐allograft rejection capabilities and prolonged graft survival in islet transplantation models by inducing the differentiation of Tregs [117]. On the contrary, Long et al. [118] revealed that this ameliorative effect on graft‐versus‐host disease was achieved independently of Tregs, instead involving the inhibition of Th1 and Th17 cells and associated proinflammatory cytokines. Furthermore, valproic acid exerts direct renal protection in IRI models, preventing dysfunction and necrosis by reducing proinflammatory cytokines [119] while simultaneously upregulating anti‐inflammatory IL‐10 [120].

Berbamine, a traditional Chinese medicine, has demonstrated immunosuppressive effects in several animal studies. It has been shown to attenuate concanavalin A‐induced immune‐mediated liver injury by significantly reducing inflammatory cytokines such as TNF‐α, INFγ, and NF‐κB [121]. Even more promisingly, berbamine has been found to significantly prolong skin allograft survival by suppressing delayed‐type hypersensitivity reactions and mixed lymphocyte reactions [122]. Collectively, these insights into potential treatment targets and predicted compounds for DGF and subsequent graft injury offer valuable avenues for future therapeutic interventions.

Molecular docking simulation has shown the possibility of a strong interaction between common immunosuppressants and three key hub DE‐IRGs. The results revealed that cyclosporin A exhibited a generally stronger binding affinity with all three molecules, followed by tacrolimus and MMF. The gold standard immunosuppressive therapy for renal transplantation employs multiple agents with distinct targets and mechanisms of action, including (1) calcineurin inhibitors, (2) mammalian target of rapamycin (mTOR) inhibitors, (3) antiproliferatives, (4) glucocorticosteroids, and (5) biological immunosuppressive agents [123]. Both cyclosporin A and tacrolimus are calcineurin inhibitors. Cyclosporin A functions by inhibiting calcineurin through the formation of a complex with the immunophilin cyclophilin, which prevents the translocation of the activated nuclear factor of activated T cells (NF‐AT). It also inhibits the activation of other TFs, such as NF‐κB, thereby preventing the induction of cytokine‐encoding genes, particularly IL‐2 [123, 124]. Tacrolimus, which is ~100 times more potent than cyclosporin A, inhibits calcineurin by binding to the immunophilin FK506 binding protein (FKBP), blocking NF‐κB‐mediated transcription of proinflammatory genes involved in T‐cell activation [125]. MMF is one of the most widely used immunosuppressive drugs, primarily inhibiting lymphocyte proliferation to prevent rejection in KTx [125, 126]. Schwarze et al. [127] demonstrated that MMF led to longer allograft survival compared to a similar course of cyclosporine in cardiac transplantation while also reducing the prevalence of cardiac allograft vasculopathy. Our study aligns with previous research, offering new mechanistic insights into the anti‐immune infiltration and rejection effects of these commonly used immunosuppressants. It is crucial to note that these drug predictions are based on in silico gene signature matching and do not equate to clinical applicability. For instance, while estradiol has shown protective effects against renal IRI in animal models [116], its systemic use in transplant recipients carries the risks of thromboembolism and hormonal disruption [128]. Similarly, acetaminophen, as identified by the algorithm, requires caution due to potential hepatotoxicity [129]. Furthermore, the potential interactions between these small molecules and standard immunosuppressive regimens (e.g., tacrolimus and MMF) remain unknown. Therefore, these findings should be viewed strictly as hypothesis‐generating, and rigorous in vivo safety and efficacy validations are mandatory before any clinical consideration. We then established a mouse UIRI model to validate our bioinformatic findings in vivo. Histological evaluation confirmed severe tubular damage and massive inflammatory cell infiltration in the injured kidneys, indicating a robust ischemic injury response. Crucially, qRT‐PCR analysis demonstrated the significant upregulation of *Bag3*, *Serpinh1*, *Klf6*, and *Slc2a3* following UIRI. *Klf6* and *Slc2a3* are strongly associated with early cellular stress and acute metabolic reprogramming during ischemia. Furthermore, the upregulation of *Bag3* and *Serpinh1* points to the early activation of profibrotic pathways. This early fibrotic signaling is a critical driver in the transition from acute ischemic injury to CKD and can significantly compromise long‐term kidney transplant prognosis. Conversely, *Nfkbia* and *Adm* showed no significant expression changes between the UIRI and Sham groups, which may be attributed to highly transient expression windows that were not captured at our specific sampling time point. Overall, the robust validation of *Bag3*, *Serpinh1*, *Klf6*, and *Slc2a3* highlights their potential as specific biomarkers and therapeutic targets for mitigating ischemic renal damage and preventing progressive fibrosis.

However, our study still possesses certain limitations. Initially, the integration of transcriptomic data from diverse platforms (microarray and RNA‐seq) introduces inherent heterogeneity due to varying experimental protocols and donor characteristics. Although we employed rigorous batch effect correction methods to mitigate technical biases, residual confounding factors may still persist. Therefore, while we meticulously screened the available datasets, the inclusion of more human posttransplant renal biopsy samples in large‐scale, standardized cohorts is necessary to minimize platform‐specific variations and enhance the robustness of our conclusions. Establishing a nomogram for prognosis prediction by integrating a broader array of clinical parameters could also improve predictive accuracy. Furthermore, despite validation in a mouse IRI model, the study lacks deep mechanistic verification. Future work will aim to establish biological causality by (1) validating signaling pathways; (2) investigating gene‐immune cell interplay (e.g., CD8+ T cells) via coculture systems; and (3) verifying regulatory networks using luciferase reporter and ChIP assays. Most importantly, regarding the drug prediction analysis, we acknowledge that our findings are currently based on in silico molecular docking and gene–drug interaction networks. While these computational predictions offer promising candidates (e.g., berbamine and estradiol), they do not equate to biological efficacy. The lack of in vivo pharmacological validation is a significant limitation of the current study. Therefore, rigorous experimental studies using relevant animal models are strictly required in the future to verify whether these small molecules can effectively modulate the identified targets, alter immune cell infiltration, and ultimately alleviate DGF and renal injury.

Therefore, our research may serve as a foundation for the development of novel diagnostic and therapeutic approaches for the management of patients post‐KTx.

## 5. Conclusion

In this study, we delved into the intricate interplay between renal IRI and allograft rejection, both posing significant threats to graft survival in KTx patients. By scrutinizing the transcriptomic landscape, we first provided novel and comprehensive insights into the immune infiltration process during the early post‐IRI stage and its consequential impact on DGF occurrence and long‐term graft survival rate. Through analysis of the expression profile of 47 DE‐IRGs, we pinpointed hub DE‐IRGs associated with DGF (ADM, SERPINH1, SLC2A3, BAG3, NFKBIA, KLF6, and CEBPD) and graft survival (BAG3, ADM, SLC2A3, SERPINH1, CEBPD, and KLF6), thereby highlighting potential molecular players in these processes. Leveraging these hub genes, we stratified post‐kidney transplant IRI patients into distinct groups, elucidating disparities in immune cell infiltration abundance and clinical characteristics among them. Additionally, we constructed transcriptional regulatory networks for DGF‐related hub DE‐IRGs, identifying the key regulatory TFs (RARA, TFDP1, SIN3A, SMC3, CHD1, MEF2A, and YY1) and miRNAs (hsa‐miR‐181a, hsa‐miR‐181b, hsa‐miR‐181c, and hsa‐miR‐181d). Potential predicted compounds were also identified (acetaminophen, estradiol, valproic acid, and berbamine), with the exploration of common immunosuppressants (cyclosporin A, MMF, and tacrolimus). These predicted compounds require further pharmacological validation. Finally, we developed predictive models for DGF (AUC = 0.832 and AUC = 0.975) and graft survival (1‐, 2‐, and 3‐year AUC = 0.773, 0.742, and 0.757) based on hub DE‐IRGs, potentially enhancing the predictive ability of transplant patients’ prognosis.

**Nomenclature**
KTx: Kidney transplantation
ESKD: End‐stage kidney disease
IRI: Ischemia‐reperfusion injury
DGF: Delayed graft function
AKI: Acute kidney injury
DAMPs: Damage‐associated molecular patterns
HSP: Heat‐shock proteins
TLRs: Toll‐like receptors
TNF: Tumor necrosis factor
APCs: Antigen‐presenting cells
Tregs: Regulatory T cells
GEO: Gene Expression Omnibus
IRGs: Immune infiltration‐related genes
DE‐IRGs: Differentially expressed immune infiltration‐related genes
IR: Ischemia‐reperfusion
IGF: Immediate graft function
PPI: Protein–protein interaction
GO: Gene Ontology
KEGG: Kyoto Encyclopedia of Genes and Genomes
ssGSEA: Single‐sample gene set enrichment analysis
RF: Random forest
SVM‐RFE: Support vector machine recursive feature elimination
LASSO: Least absolute shrinkage and selection operator
IC: Item‐consensus
CLC: Cluster‐consensus
ROC: Receiver operating characteristic
DI‐ICs: Differentially infiltrated immune cells
TFs: Transcription factors
miRNAs: MicroRNAs
DSigDB: Drug Signatures Database
MMF: Mycophenolate mofetil
AUC: Area under the curve
PH: Proportional hazard.

## Author Contributions

Conceptualization: Yifei Zhang, Yuqing Li, Xuemeng Qiu, Jiyue Wu, Qing Bi, Jiandong Zhang, and Wei Wang. Methodology: Yifei Zhang, Yuqing Li, Xuemeng Qiu, Jiyue Wu, Qing Bi, and Jiandong Zhang. Software, investigation: Yifei Zhang, Yuqing Li, Xuemeng Qiu, and Jiyue Wu. Validation: Yifei Zhang, Xuemeng Qiu, and Peng Cao. Data curation: Yifei Zhang, Yuqing Li, and Qing Bi. Formal analysis: Yifei Zhang, Yuqing Li, Xuemeng Qiu, and Jiyue Wu. Resources: Yifei Zhang, Yuqing Li, Xuemeng Qiu, Jiyue Wu, and Qing Bi. Visualization: Yifei Zhang and Xuemeng Qiu. Writing – original draft preparation: Yifei Zhang. Writing – review and editing: Yuqing Li, Xuemeng Qiu, Jiyue Wu, Qing Bi, Peng Cao, Jiandong Zhang, and Wei Wang. Supervision, project administration: Jiandong Zhang and Wei Wang. Funding acquisition: Wei Wang.

## Funding

The study was supported by the National Natural Science Foundation of China (Grant 82070764).

## Disclosure

All authors have read and agreed to the published version of the manuscript.

## Ethics Statement

This study analyzed publicly available, deidentified transcriptomic datasets obtained from public repositories and did not involve direct contact with human participants or the collection of new human specimens. Therefore, additional ethical approval for the use of human data and individual informed consent were not required. The animal study protocol was approved by the Institutional Animal Care Ethics Committee of Beijing Chaoyang Hospital on February 28, 2023 (Ethics Number 2023‐animal‐97). All surgical procedures, animal handling, and husbandry were performed in accordance with the approved institutional guidelines.

## Conflicts of Interest

The authors declare no conflicts of interest.

## Supporting Information

Additional supporting information can be found online in the Supporting Information section.

## Supporting information


**Supporting Information 1** Table S1. Summary of Gene Expression Omnibus (GEO) series datasets. Table S2. List of immune infiltration‐related genes retrieved from GeneCards. Table S3. Identification of differentially expressed immune infiltration‐related genes (DE‐IRGs). Table S4. Calculation of coefficients for DGF‐related hub DE‐IRGs. Table S5. Interaction network analysis of transcription factors (TFs) and hub DE‐IRGs. Table S6. Regulatory network results for TFs, miRNAs, and hub DE‐IRGs. Table S7. Predicted therapeutic drugs via Enrichr analysis. Table S8. Prognostic analysis and coefficients for survival‐related hub DE‐IRGs. Table S9. Genes and primer sequences.

**Supporting Information 2** Figure S1. Consensus clustering results based on various *k* values (*k* = 2 to *n*).

## Data Availability

The datasets analyzed during the current study are publicly available in the GEO database. The specific accession numbers for all utilized GEO datasets are detailed in Supporting Information 1: Table S1. The custom R analysis scripts used to support the findings of this study are available from the corresponding author upon reasonable request.
