# Supplementary figures and images for: Immune Infiltration‐Related Genes as Potential Biomarkers and Predicted Targets for Renal Allograft Delayed Graft Function and Survival Outcome: An Integrated Machine Learning Approach and Drugs Analysis
Source: Mediators Inflamm. 2026 Jun 12;2026:1451740. doi: 10.1155/mi/1451740 (PMC13261374; doi:10.1155/mi/1451740)

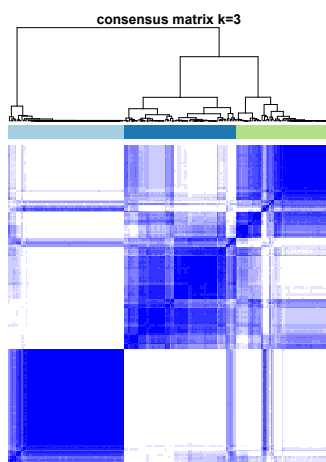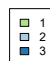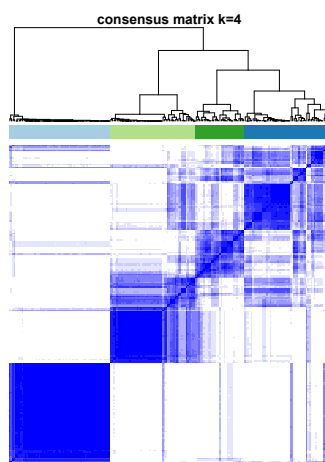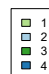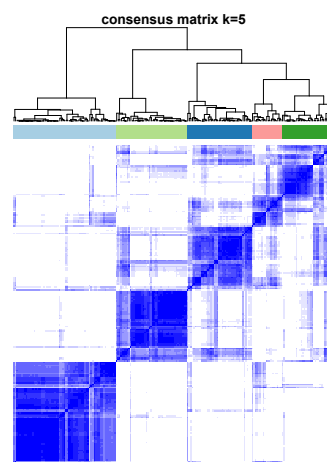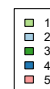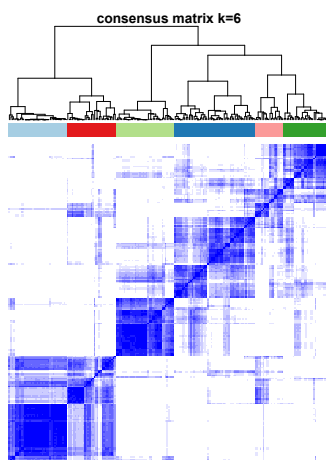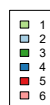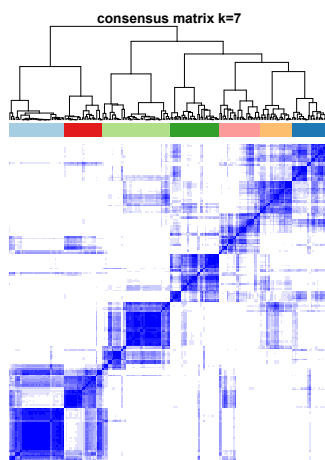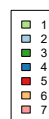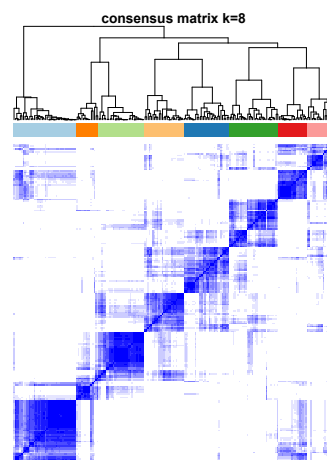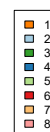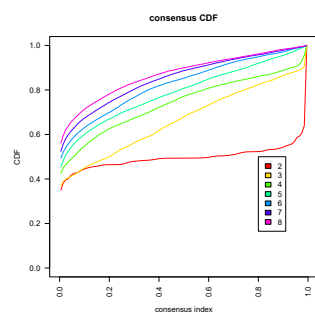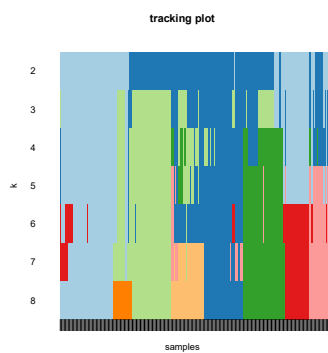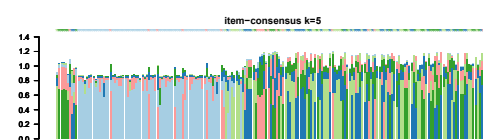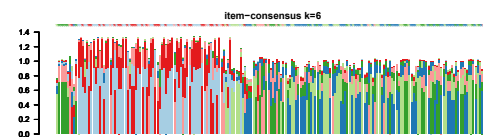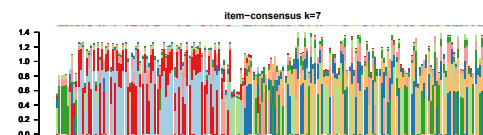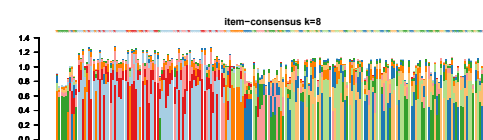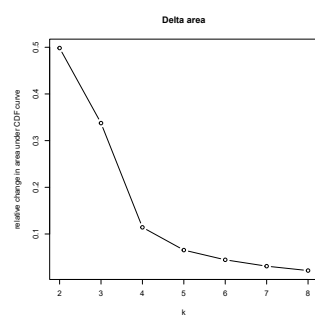

Supplement: Supplementary file 2 — Supporting Information 2 Figure S1. Consensus clustering results based on various k values (k = 2 to n). [file MI-2026-1451740-s002.pdf]
